# scTACL: a multitask topology-aware contrastive learning approach for single-cell transcriptomics analysis

**DOI:** 10.1093/bioinformatics/btag361

**Published:** 2026-06-04

**Authors:** Murong Zhou, Xin Lu, Yingjian Liang, Alfred Wei Chieh Kow, Guohua Wang, Qiaoming Liu, Yuming Zhao

**Affiliations:** School of Computer Science and Artificial Intelligence, Northeast Forestry University, Harbin 150040, China; School of Computer Science and Artificial Intelligence, Northeast Forestry University, Harbin 150040, China; Hepatobiliary and Pancreatic Surgery, Harbin Medical University Cancer Hospital, Harbin 150080, China; Division of Hepatobiliary & Pancreatic Surgery, Department of Surgery, National University Hospital, Singapore, 119074, Singapore; Department of Surgery, Yong Loo Lin School of Medicine, National University of Singapore, Singapore, 119228, Singapore; School of Computer Science and Artificial Intelligence, Northeast Forestry University, Harbin 150040, China; Department of Computer Science and Technology, Faculty of Computing, Harbin Institute of Technology, Harbin 150001, China; School of Artificial Intelligence, Henan University, Zhengzhou 450000, China; School of Computer Science and Artificial Intelligence, Northeast Forestry University, Harbin 150040, China

## Abstract

**Motivation:**

The advent of single-cell RNA sequencing (scRNA-seq) technology has allowed researchers to measure gene expression profiles at the single-cell level, providing valuable insights into cellular heterogeneity. However, due to the limitations of current sequencing platforms, scRNA-seq data often contain significant noise, particularly severe dropout events, which pose major challenges for subsequent analyses.

**Results:**

In this study, we developed a new method called topology-aware contrastive learning (scTACL). This approach uses contrastive learning between a cell similarity graph and a cell embedding similarity graph, employing a zero-inflated negative binomial (ZINB) distribution to model the reconstructed data. This alignment helps the processed data better reflect true biological signals. It delivers superior results in key tasks such as data imputation, clustering, batch effect correction, and cell–cell interaction. Additionally, scTACL successfully identified two distinct subtypes of epithelial cells in lung adenocarcinoma tissues, further demonstrating its effectiveness and usefulness in complex biological settings. Notably, without relying on spatial location information, scTACL still effectively distinguished the epithelial and mesenchymal regions in the spatial transcriptome data of liver cancer and identified the COLLAGEN signaling pathway, which plays a crucial role in the epithelial–mesenchymal transition process through intercellular communication analysis.

## 1 Introduction

As fundamental units of life, cells perform a variety of biological functions depending on their tissue context and physiological state. Although traditional bulk RNA sequencing offers high sequencing depth, it captures only the average transcriptional profile of large cell populations, making it challenging to uncover cell-to-cell heterogeneity (Mortazavi *et al*. 2008). Recently, groundbreaking advances in single-cell RNA sequencing (scRNA-seq) have enabled the precise profiling of gene expression at the individual-cell level (Tang *et al*. 2009). This opportunity has significantly deepened our understanding of cellular heterogeneity. In addition to transforming research approaches in cell biology and epigenetics, scRNA-seq has also profoundly influenced the analysis of complex biological systems, such as the tumor microenvironment (Han *et al*. 2023).

Although scRNA-seq has made groundbreaking progress in elucidating cellular heterogeneity, it still faces substantial challenges. At the technical level, an inherent trade-off exists between sequencing sensitivity and throughput. For example, high-sensitivity platforms (such as Smart-seq2) can thoroughly capture transcripts, especially low-abundance genes and noncoding RNAs, but they have limited sample capacity and higher costs (Picelli *et al*. 2013). Conversely, high-throughput platforms (e.g. Drop-seq) are ideal for large-scale cell sequencing but often sacrifice sequencing depth per cell (Macosko *et al*. 2015). Additionally, systematic biases introduced by different sequencing platforms or experimental batches—known as batch effects—can obscure true biological signals or lead to false-positive results, thereby compromising the accuracy of biological interpretations.

A more significant issue is the high dimensionality and sparsity of scRNA-seq data, particularly evident in dropout events, where gene expressions that should be detected remain undetected due to technical limitations. To mitigate these effects, various computational strategies have been proposed. In the context of scRNA-seq, “denoising” refers to the reduction of technical noise introduced during sequencing and library preparation, aiming to improve data fidelity by removing random fluctuations. In contrast, “imputation” focuses on the recovery of dropout (zero-inflated) expression values resulting from low-capture efficiency. While both strategies enhance data quality, they target distinct sources of data distortion. The data sparsity caused by dropouts significantly impacts downstream analyses in several ways: (i) Cell clustering, a key step in scRNA-seq analysis pipelines, heavily relies on the quality of the raw data. The high dropout rate-induced sparsity significantly reduces clustering accuracy. (ii) Dropouts can obscure real gene expression differences, lowering sensitivity and increasing the risk of false positives. This effect is especially pronounced for genes with low expression, which may include critical regulatory factors and cell-specific markers. (iii) Cell type identification depends on accurately detecting marker genes. When marker genes are missing because of dropout, misidentification is more likely, particularly among transcriptionally similar subtypes. (iv) The coexpression of ligand–receptor pairs is fundamental for inferring cell–cell communication. Loss of gene expression can lead to the missed detection of key signaling pathways, thereby affecting the comprehensive understanding of cellular interactions and tissue function.

Regarding dropout issues, numerous approaches for imputing scRNA-seq data have recently emerged, with notable methods including SAVER (Huang *et al*. 2018), scImpute (Li and Li 2018), and MAGIC (Van Dijk *et al*. 2018). Specifically, SAVER employs Bayesian regression and coexpression networks, leveraging gene similarities to build predictive models that estimate the true expression level of each gene in individual cells. In contrast, scImpute initially clusters cells into subgroups and assesses whether zero values result from dropout events based on expression patterns within the same cluster. MAGIC, grounded in manifold learning theory, constructs cell similarity graphs and employs diffusion processes to propagate information across the graph, smoothing and completing the gene expression matrix. With rapid advances in deep learning, driven by their strong learning capabilities and scalability, some researchers have developed deep learning-based methods for denoising single-cell transcriptomics data. For instance, scVGAE (Inoue 2024) integrates a graph convolutional network (GCN; Kipf and Welling 2017) into a variational autoencoder (VAE) framework (Kingma and Welling 2014), using the zero-inflated negative binomial (ZINB) loss function to achieve precise data recovery. AcImpute (Zhang *et al*. 2025) assumes that lower gene expression levels within the same cell type correlate with higher dropout rates. It builds a transition probability matrix between cells and recovers missing expression values through the weighted reconstruction of this matrix.

For the cell clustering issues, Seurat (Stuart *et al*. 2019) and Scanpy (Wolf *et al*. 2018) represent two of the most prominent toolkits in R and Python, respectively. Seurat combines scRNA-seq data with in situ RNA patterns to infer cell locations and types, whereas Scanpy is a scalable toolkit for analyzing scRNA-seq gene expression data built with anndata. Both tools utilize a shared nearest neighbor model and either the Leiden or Louvain algorithms for clustering. In addition to these traditional tools, deep learning techniques have been increasingly applied to enhance clustering performance in scRNA-seq analysis. scSimGCL utilizes multi-head attention mechanisms to integrate gene expression and graph structure information, extracting features through graph neural networks) to achieve clustering (Zhang *et al*. 2024). GraphSCC combines a graph GCN with denoising autoencoders and optimizes the model through a dual self-supervised module to enhance clustering performance (Zeng *et al*. 2020).

**Figure 1:**
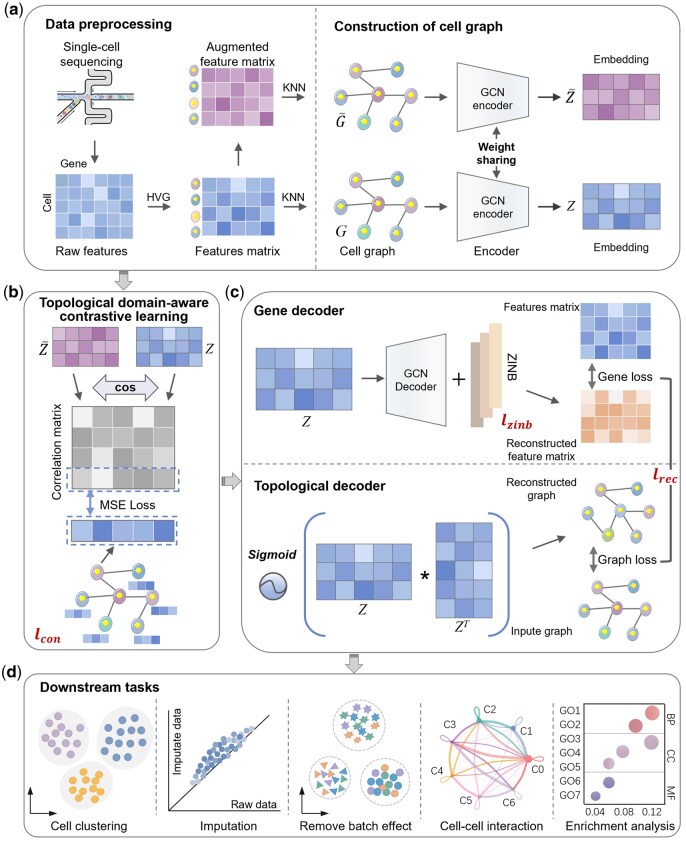
Overview of scTACL. (a) Following the selection of HVGs, scTACL constructs the original graph G by integrating gene expression profiles and the Euclidean distances computed in the PCA space using the K-nearest neighbors (KNN) algorithm. Subsequently, a data augmentation strategy is applied to the original graph G to generate a corresponding augmented graph $\tilde{G}$. Finally, scTACL takes both the original graph G and the augmented graph $\tilde{G}$ as inputs to the model and produces the corresponding intermediate embeddings Z and $\tilde{Z}$. (b) scTACL is introduced to enhance the model’s ability to capture cell-cell relationships in the latent space. (c)The gene decoder adopts a symmetric architecture to the encoder, while the topology decoder employs an inner product decoder and leverages a ZINB (Zero-Inflated Negative Binomial) model to fit the reconstructed gene expression matrix. (d) Downstream analysis tasks were conducted on the reconstructed matrix obtained by scTACL.

Inspired by these advances, we propose a topology-aware contrastive learning (scTACL framework; Fig. 1). scTACL is a unified model that supports multiple downstream analyses, including data imputation, cell clustering, batch effect correction, differential expression analysis, trajectory inference, and cell–cell interaction/communication analysis. Unlike conventional pipelines that perform imputation and clustering as separate, sequential steps, scTACL directly reuses the imputed expression matrix produced during training as a shared representation for all downstream tasks. This design improves interpretability and simplifies the workflow by reducing inter-task dependencies and minimizing ad hoc preprocessing.

In terms of modeling strategy and learning objectives, scTACL differs fundamentally from existing GCN- or ZINB-based approaches. First, beyond reconstructing gene expression alone, scTACL introduces a topological decoder that jointly reconstructs gene expression and cell–cell graph structure. This strategy encourages structural consistency between latent representations and biological neighborhood relationships. Second, scTACL incorporates a topology-aware contrastive objective that aligns representations learned from the original and augmented graphs while explicitly preserving topological constraints. In contrast, most conventional GCN- or VAE-based models rely primarily on reconstruction losses and do not explicitly regularize the latent space with scTACL. Together, these components enable scTACL to jointly model gene expression, cellular topology, and representation robustness within a single framework, thereby providing a streamlined paradigm for multifaceted single-cell transcriptome analysis.

To comprehensively evaluate scTACL, we conducted systematic experiments on 20 single-cell and spatial transcriptomics datasets spanning diverse tissues and sequencing platforms, including 17 benchmark scRNA-seq datasets for quantitative evaluation and 3 datasets for downstream analyses. The tasks evaluated included data imputation, cell clustering, batch effect correction, differential expression analysis, pseudotime trajectory inference, and cell–cell communication prediction, offering comprehensive and multidimensional validation of the method’s effectiveness. The results show that scTACL consistently surpasses leading state-of-the-art methods in key areas such as imputation, clustering, and batch effect removal, demonstrating superior accuracy and robustness. In differential expression analysis, scTACL successfully identified transcriptional differences between distinct epithelial subtypes in a lung adenocarcinoma (LUAD) dataset, underscoring its ability to detect subtle cellular variations. Notably, even without spatial location data, scTACL was able to accurately infer potential epithelial–mesenchymal transition (EMT) trajectories in a hepatocellular carcinoma spatial transcriptomics dataset, underscoring its strong potential to capture complex spatial–functional relationships within biological systems.

## 2 Methods

### 2.1 Data preprocessing

In this study, we begin with the raw scRNA-seq gene expression count matrix, denoted as $X_{\mathrm{Raw}}\in R^{N\times M}$, where *N* represents the number of cells and *M* represents the number of genes. Owing to the high-dimensional sparsity inherent in scRNA-seq data, directly inputting the full expression matrix into the model would not only incur excessive memory usage but also introduce unnecessary noise. To address this issue, we utilize the *Scanpy* toolkit to perform highly variable gene (HVG) selection on the raw matrix. This application results in a reduced matrix of HVG expressions, denoted as $X_{\mathrm{HVG}}\in R^{N\times H}$, where *H* is the number of selected HVGs. Then, we apply a standardized preprocessing pipeline to $X_{\mathrm{HVG}}$, including normalization to unit variance and a zero mean across genes. The resulting normalized gene expression matrix is denoted as $X\in R^{N\times H}$, which serves as the input to the downstream components of our model.

### 2.2 Construction of the cell graph

Unlike previous studies that rely solely on the scRNA-seq gene expression matrix as input, we introduce an additional graph structure to capture the underlying relationships between cells more effectively. To this end, we develop a graph construction approach based on principal component analysis (PCA) and K-nearest neighbors (KNN), enabling a more accurate representation of cell–cell interactions. Specifically, we first apply PCA to the normalized gene expression matrix $X\in R^{N\times H}$, resulting in a low-dimensional principal component matrix $X_{\mathrm{PCA}}\in R^{N\times D}$, where each cell is represented by a *D*-dimensional vector. In the reduced space, we compute the Euclidean distance between pairs of cells to quantify their similarity: The smaller the distance between two cells, the more similar their gene expression profiles are in the PCA-transformed space. Next, we apply the KNN algorithm to identify the top *K* nearest neighbors for each cell, constructing a binary adjacency matrix $A\in R^{N\times N}$, where $A_{ij}=1$ if cell $v_{i}$ is among the top *K* nearest neighbors of cell $v_{j}$, and $A_{ij}=0$ otherwise. The resulting graph structure is denoted as G=(V,E), where $V=(v_{1},v_{2},\ldots v_{N})$ represents the set of nodes (cells); and where E⊆V×V represents the set of edges derived from the adjacency matrix A.

### 2.3 Graph convolutional autoencoder combined with the ZINB model

To capture the latent structure in scRNA-seq data more effectively, we employ a GCN autoencoder that integrates gene expression features with the topological structure of cell–cell relationships. Additionally, to address the highly sparse nature of scRNA-seq data, we incorporate a ZINB model to approximate their underlying distribution better. Specifically, the encoder and decoder are implemented as single-layer symmetric graph convolutional networks (GCNs). The encoder learns low-dimensional cell embeddings by propagating gene expression signals through the cell graph, whereas the decoder aims to reconstruct the input representation. To enhance the reconstruction fidelity, we append a ZINB module to the output of the decoder. This probabilistic component enables the model to capture the overdispersed nature of scRNA-seq counts and the excess of zero values resulting from dropout events, thereby yielding a more biologically realistic representation of the data distribution.

The graph structure *G* is composed of the normalized gene expression matrix X and the adjacency matrix *A*, which together serve as the input to the encoder. The encoder maps this input to a latent embedding *Z*, capturing feature and structural information. The decoder then uses *Z* to reconstruct the gene expression matrix, denoted as *H*. This process can be formally described as follows:


(1) $$Z=\;\sigma (\tilde{A}XW_{e}+B_{e})$$

(2) $$H=\;\sigma (\tilde{A}ZW_{d}+B_{d})$$

(3) $$\tilde{A}=\;D^{-\frac{1}{2}}\hat{A}D^{-\frac{1}{2}}$$

where $\hat{A}=A+I$ is the adjacency matrix with added self-loops; $D=\sum_{j}A_{ij}$ is the degree matrix; $W_{e}$ and $W_{d}$ are the trainable weight matrices in the encoder and decoder, respectively; $B_{e}$ and $B_{d}$ are the corresponding bias vectors for the encoder and decoder; and σ(·) denotes a nonlinear activation function (such as RELU).

After obtaining the reconstructed matrix *H*, we model it via the ZINB distribution to more accurately reflect the statistical properties of the scRNA-seq data, which are typically characterized by overdispersion and an excess of zero counts (dropouts). The ZINB model models the distribution of gene expression by fitting three parameters: the mean μ, dispersion θ, and dropout rate π, which are defined as follows:


(4) $$\mu =\mathrm{diag}(S_{i})\times \exp\left(W_{\mu }H\right)$$

         $\theta =\exp\left(W_{\theta }H\right)$(5)


(6) $$\pi =\sigma (W_{\pi }H)$$

the ZINB distribution function is defined as follows:


(7) $$\mathrm{ZINB}(X|\pi ,\mu ,\theta )=\pi \delta (X_{m})+(1-\pi )[\frac{\Gamma (X+\theta )}{X!\Gamma (\theta )}{\left(\frac{\theta }{\theta +\mu }\right)}^{\theta }{\left(\frac{\mu }{\theta +\mu }\right)}^{X_{m}}]$$

In addition to reconstructing the gene expression matrix, we introduce an inner product decoder to reconstruct the cell adjacency matrix *A*. The reconstructed adjacency matrix output by the inner product decoder is denoted as $\hat{A}$, which is defined as follows:


(8) $$\hat{A}=\mathrm{sigmoid}(Z^{T}Z)$$

where $\mathrm{sigmoid}(x)=1/(1+e^{-x})$ is a nonlinear activation function.

To capture gene expression patterns and intercellular relationships simultaneously, we optimize the model by jointly minimizing the reconstruction errors of the gene expression matrix and the adjacency matrix. The overall reconstruction loss is defined as follows:


(9) $$l_{\mathrm{rec}}=\frac{1}{N}\sum_{i=1}^{N}\left({\alpha_{1}\|H_{i}-X_{i}\|}^{2}+{\alpha_{2}\|{\hat{A}}_{i}-A_{i}\|}^{2}\right)$$

where N is the number of cells; *i* indexes the *i*-th cell; and $\alpha_{1}$ and $\alpha_{2}$ are balancing coefficients between gene expression and graph reconstruction loss. Through empirical testing, we find that the model achieves optimal performance when $\alpha_{1}=1.0$ and $\alpha_{2}=0.6$. In addition to reconstruction loss, we also incorporate ZINB-based loss to model the overdispersed and zero-inflated nature of scRNA-seq data. This loss is defined as follows:


(10) $$l_{\mathrm{zinb}}=\sum -\log(\mathrm{ZINB}(X|\pi ,\mu ,\theta ))$$

The ZINB parameters (μ, θ, π) are obtained by applying separate fully connected layers with appropriate activation functions to the decoder output H.

### 2.4 Topological domain-aware contrastive learning

To increase the quality of latent embeddings, we propose a scTACL. In each iteration, the framework takes two graphs as inputs: the original graph *G*, constructed from the normalized matrix *X* and its adjacency matrix *A*, and an augmented graph $\tilde{G}$, derived from the original graph through data augmentation. Specifically, for the original graph *G*, we perturb the node features by shuffling the normalized gene expression matrix *X w*hile keeping the topological structure (i.e., adjacency matrix *A*) unchanged. This undertaking results in an augmented graph structure $\tilde{G}=(V,E)$, where $\tilde{X}$ is the shuffled version of *X*, and *A* remains fixed. This approach improves the model’s ability to discern spurious noise, enhances its robustness to diverse features, and further explores the latent information in scRNA-seq data. The original graph *G* and the augmented graph $\tilde{G}$ are fed into a shared-parameter GCN encoder to generate distinct latent embeddings *Z* and $\tilde{Z}$, where the computation of $\tilde{Z}$ can be expressed as follows:


(11) $$\tilde{Z}=\;\sigma (\tilde{A}\tilde{X}W_{e}+B_{e})$$

where $\tilde{A}$, $W_{e}$, $B_{e}$ and σ(·) are the same as those in Equation (1).

Although Equations (1) and (11) are mathematically identical in form, they operate on different graph inputs. Specifically, Equation (1) processes the original graph G=(X,A), where X denotes the normalized gene expression matrix. Equation (11), in contrast, processes the augmented graph $G^{\prime }=(X^{\prime },A)$, where $X^{\prime }$ is generated by randomly perturbing the node features while retaining the original adjacency matrix A. This design enables the contrastive learning module to learn representations that are robust to feature perturbations while preserving the underlying cell–cell relational structure.

Contrastive learning aims to maximize the similarity between positive samples while minimizing the similarity between negative samples. To this end, we propose a scTACL, which assumes that cells within the same class exhibit greater similarity, whereas cells from different classes have lower similarity. Based on this reasonable assumption, we calculate the cosine similarity matrix of cross-view latent embeddings, defined as follows:


(12) $$S_{ij}=\frac{(Z_{i}){\left({\tilde{Z}}_{j}\right)}^{T}}{\left\|Z_{i}\|\|{\tilde{Z}}_{j}\right\|}$$

where $S_{ij}$ represents the cosine similarity between the embedding $Z_{i}$ of the *i*-th sample in the original view and the embedding ${\tilde{Z}}_{j}$ of the same sample in the augmented view. Since the graph topology is also constructed based on the similarities between cells, we introduce a mean square error (MSE) loss to make the similarity matrix *S* as close as possible to the adjacency matrix *A*. The MSE loss is defined as follows:


(13) $$l_{\mathrm{con}}=\frac{1}{N^{2}}\sum_{i=1}^{N}\sum_{j=1}^{N}{\left(S_{ij}-A_{ij}\right)}^{2}$$

where *N* is the number of cell samples. Through MSE loss, during model training, the similarity between point pairs in the latent embeddings increases when $A_{ij}=1$ and decreases otherwise. This process enables scTACL to effectively pull closer points with high similarity while pushing apart points with low similarity. Consequently, the latent embeddings can efficiently capture the complex relationships hidden in the data, considerably improving the quality of the learned representations.

### 2.5 Overall loss function

To enhance the overall performance of the model, we adopt a joint optimization strategy during the training process, which integrates reconstruction loss, ZINB loss, and scTACL loss. The overall objective loss function is defined as follows:


(14) $$L=\alpha *l_{\mathrm{rec}}+\beta *l_{\mathrm{zinb}}+\gamma *l_{\mathrm{con}}$$

where $l_{\mathrm{rec}}$ represents the reconstruction loss, $l_{\mathrm{zinb}}$ represents the ZINB loss, and $l_{\mathrm{con}}$ represents the scTACL loss. The coefficients α,β, and γ are weight-balancing parameters that control the contribution of each component.

Although scTACL incorporates a ZINB probabilistic model during training, the final imputed gene expression matrix used for downstream analyses is the deterministic reconstruction H. Specifically, the ZINB loss acts as a probabilistic regularization term that encourages the model to capture biologically realistic count distributions, including overdispersion and dropout events commonly observed in single-cell transcriptomic data. In contrast, the deterministic reconstruction loss focuses on preserving the global structure of the gene expression space and stabilizing the learned latent representation. By combining these two objectives, the model can simultaneously learn robust feature representations while maintaining consistency with the statistical characteristics of the observed count data.

Importantly, using Hto parameterize the ZINB distribution does not introduce conflicts. The reconstruction loss does not enforce exact fitting of zero entries but instead serves as a regularization mechanism that promotes stable embeddings and smooth reconstructions, whereas the ZINB likelihood explicitly models dropout uncertainty through its zero-inflation component. As a result, the probabilistic modeling of gene expression counts is primarily governed by the ZINB objective, while the reconstruction term improves representation stability and training robustness. Similar hybrid optimization strategies that combine deterministic reconstruction with probabilistic count modeling have been successfully adopted in several deep learning frameworks for single-cell data analysis, such as scVGAE, scE2EGAE (Wang *et al*. 2025), ZMGA (Yao *et al*. 2024), and scZiva (Vo *et al*. 2026), demonstrating the effectiveness of integrating representation learning with count-based statistical modeling.

Together, these objectives enable scTACL to achieve a balance between statistical realism and practical usability, leading to improved representation quality and more reliable downstream analyses.

Taking clustering as an example, after the reconstructed gene expression matrix *H* is obtained, dimensionality reduction is performed via PCA. For datasets with a known number of clusters, the K-means algorithm is applied, with the number of clusters specified accordingly. For datasets with an unknown number of clusters, the Leiden clustering algorithm from the Scanpy package is employed, with the resolution parameter selected from the range [0.1, 0.3, 0.5]. This approach not only achieves effective imputation but also enables the precise classification of cell types, thereby uncovering potential mechanisms underlying the differences between various cell types.

### 2.6 Parameter settings

In this experiment, we define the encoder and decoder as single-layer GCN networks, with an intermediate embedding dimension of 96. The number of HVGs is set to 3000, and the number of neighbors is set to 5. The three weight-balancing parameters in the loss function, α, β, and γ, are empirically set to 5, 0.6, and 0.6, respectively. This configuration prioritizes the stability of the reconstructed representation while still incorporating probabilistic modeling of gene expression counts. A relatively larger weight is assigned to the reconstruction loss to ensure that the decoder preserves the global structure of the gene expression space and produces stable embeddings for downstream analyses. Meanwhile, the ZINB loss, although assigned a smaller weight, provides an important probabilistic constraint for modeling overdispersion and dropout events commonly observed in single-cell transcriptomic data. In practice, this weighting strategy leads to more stable training behavior and improved clustering performance across multiple datasets. Therefore, the chosen parameter setting allows scTACL to effectively balance representation learning and distribution-aware modeling.

The optimizer used is the Adam algorithm with a learning rate of 0.0008 and no decay. The training runs for 500 iterations. Additionally, the experiment is conducted on a server equipped with an NVIDIA Tesla A100 GPU. In addition to the settings reported above, detailed parameter values and implementation configurations are provided in the Additional file 1: Table 1, available as supplementary data at *Bioinformatics* online to facilitate reproducibility.

### 2.7 Benchmark experiments

To fully verify the performance of scTACL, we conducted a comprehensive comparison with existing methods, including data imputation performance, clustering performance, and batch effect removal performance. The parameters of the benchmark methods were set according to the parameters provided in their original papers (Additional file 2: Table 2, available as supplementary data at *Bioinformatics* online).

### 2.8 Benchmarking evaluation metrics for scTACL

In this study, we adopted two widely used evaluation indicators, namely normalized mutual information (NMI) and adjusted rand index (ARI), to evaluate the clustering performance of scTACL.

NMI measures the quality of clustering by calculating the NMI between the predicted label and the true label, with a value ranging from 0 to 1. The higher the NMI value, the higher the similarity between the predicted label and the true label. NMI is defined as follows:


(15) $$\mathrm{NMI}(U,V)=\frac{MI(U,V)}{\sqrt{H(U)H(V)}}$$

here, *U* and *V* correspond to the true labels and clustering results, respectively. MI(·) denotes mutual information, and H(·) denotes entropy.

ARI is a modified version of the Rand Index, widely used to assess the similarity between two clustering results. Its values range from −1 to 1, where a value closer to 1 indicates higher clustering accuracy, and a value of 0 suggests random label assignment. The ARI is calculated using the following formula:


(16) $$\mathrm{ARI}(U,V)=\frac{RI(U,V)-E[RI(U,V)]}{\max(RI(U,V)-E[RI(U,V)])}$$

where E[·] denotes expectation, and RI(·) represents the similarity between two clustering results. The calculation formula is shown below:


(17) $$RI(U,V)=\frac{TP+TN}{TP+TN+FP+FN}$$

where TP is true positive, TN is true negative, FP is false positive, and FN is false negative.

In addition to the above two indicators, we also introduced the Average Silhouette Width (ASW) to evaluate the experimental effect from multiple perspectives. ASW is often used to measure the rationality of clustering of samples within their clusters and the degree of separation from other clusters.

For each sample i, its silhouette score s(i) is defined as:


(18) $$s(i)=\frac{b(i)-a(i)}{\max(a(i)-b(i))}$$

where a(i) is the average distance between sample i and all other samples within the same cluster; b(i) is the average distance between sample i and all samples in the nearest neighboring cluster.

The silhouette score ranges from −1 to 1, where values close to 1 indicate that the sample is well clustered, close to 0 suggest that the sample lies at the boundary between clusters, and values close to −1 imply possible misclassification. The ASW is defined as:


(19) $$\mathrm{ASW}=\frac{1}{N}\sum_{i=1}^{N}s(i)$$

where N is the total number of samples. A higher ASW value indicates a clearer and more meaningful clustering structure.

## 3 Results

### 3.1 The imputed matrix of scTACL can enhance clustering performance on cross-platform datasets

**Figure 2:**
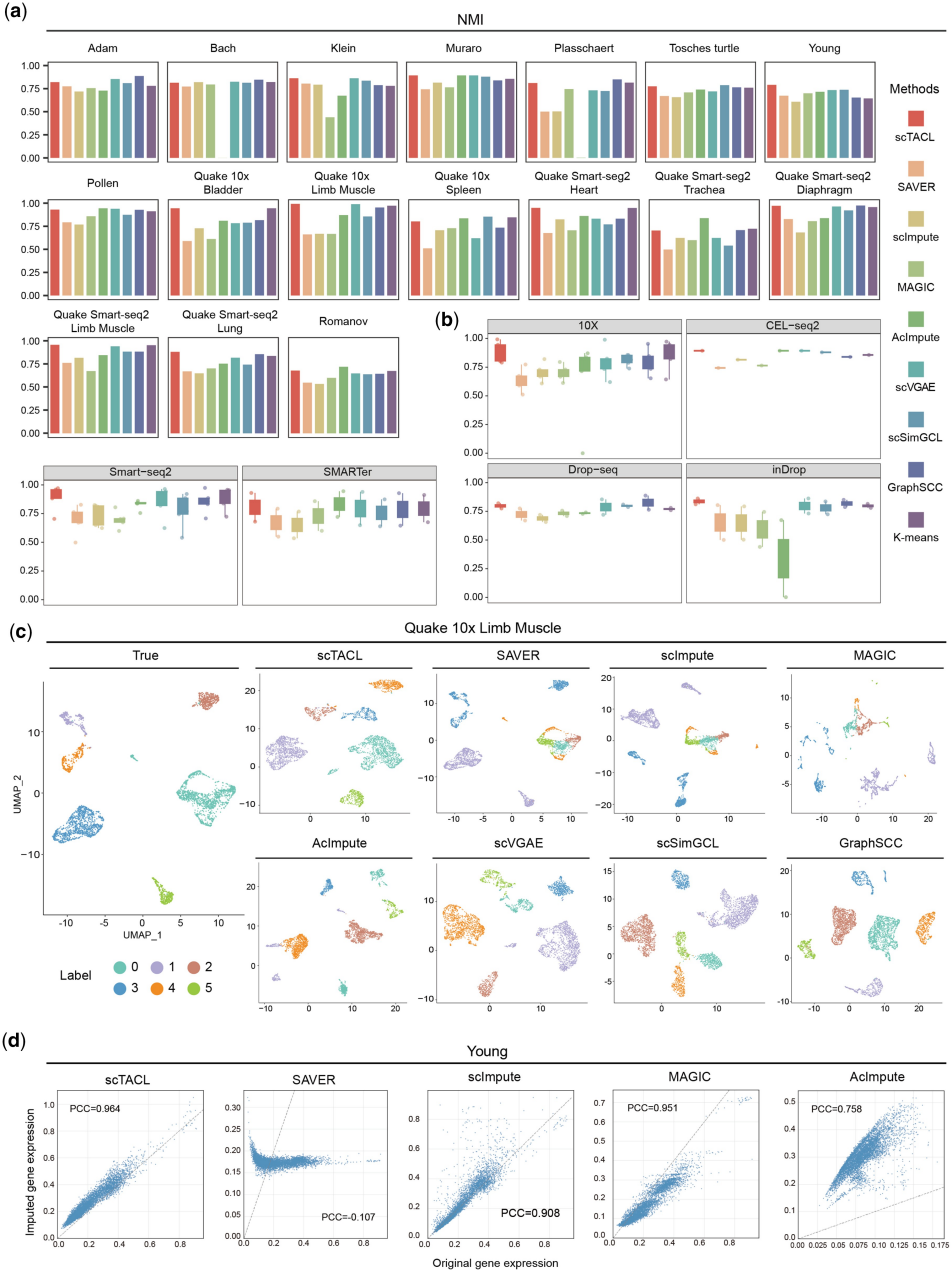
Clustering and imputation performance of scTACL. (a) NMI scores of clustering results for scTACL and baseline methods across 17 datasets. (b) The clustering performance of scTACL and benchmark methods on different sequencing platforms was evaluated and compared using the NMI metric. (c) Comparison of clustering results with 2D visualization by UMAP on the “Quake 10x Limb Muscle” dataset. (d) Data imputation performance of scTACL and other methods on the Young dataset.

To thoroughly evaluate the clustering performance of scTACL, we performed comparison experiments on 17 cross-platform scRNA-seq datasets (Additional file 2: Table 3, available as supplementary data at *Bioinformatics* online) to assess its ability to cluster effectively. The benchmark methods include models mainly designed for clustering tasks (e.g. scVGAE, scSimGCL, GraphSCC, and K-means) as well as models focused on imputation tasks (e.g. SAVER, scImpute, MAGIC, and AcImpute). Because these datasets contain ground-truth labels, we used common metrics, the NMI and ARI, to measure clustering performance (Fig. 2a and Additional file 3: Tables 4 and 5, available as supplementary data at *Bioinformatics* online). Notably, AcImpute was excluded from the Bach dataset comparison because it did not finish within 24 h.

The experimental results show that scTACL outperforms other methods on 11 out of 17 datasets and ranks among the top three on 16 of these datasets. Additionally, the average NMI and ARI values for scTACL across all datasets reached 0.8511 and 0.8277, with medians of 0.8418 and 0.8378, respectively, both notably higher than those of the other method.

To further validate the cross-platform generalization ability of scTACL, we compared its overall clustering performance across various data platforms, including 10× (Zheng *et al*. 2017), CEL-seq2 (Hashimshony *et al*. 2016), Drop-seq (Macosko *et al*. 2015), InDrop (Klein *et al*. 2015), Smart-seq2 (Picelli *et al*. 2013), and SMARTer (Ramsköld *et al*. 2012). The results show that scTACL achieved significantly better clustering performance across multiple platforms, demonstrating its efficiency and accuracy in cross-platform data processing (Fig. 2b). Additionally, we visualized the dimensionality reduction and clustering results of scTACL and other methods using UMAP. For example, in the Quake 10× Limb Muscle dataset, scTACL exhibited superior cell clustering performance. Compared with other models, scVGAE, scSimGCL and GraphSCC also produced relatively good clustering results (Fig. 2c), which is consistent with their high NMI scores (Additional file 3: Table 4, available as supplementary data at *Bioinformatics* online). Additional file 4: Fig. 1, available as supplementary data at *Bioinformatics* online presents the UMAP clustering results of scTACL on datasets other than the Quake 10× Limb Muscle dataset. The visualizations highlight that scTACL consistently achieves robust clustering performance across multiple datasets, indicating its ability to effectively preserve the underlying information and distribution patterns in gene expression matrices, thereby excelling in clustering tasks.

### 3.2 scTACL can effectively impute scRNA-seq data under high noise conditions

To systematically assess the performance of various methods in single-cell data imputation, we compared the proposed scTACL method with other leading imputation algorithms using the 16 public datasets mentioned above. Figure 2d illustrates the performance of each method on the Young dataset (results for the other datasets are presented in Additional file 5: Figs. 2–4, available as supplementary data at *Bioinformatics* online). In this figure, each point represents a single cell, with the *x*-axis indicating the original mean gene expression and the *y*-axis indicating the mean expression after imputation, thereby reflecting the correlation between them. We used the Pearson correlation coefficient (PCC) as a quantitative metric, where higher PCC values indicate that the imputed results more closely match the true expression levels, demonstrating better imputation quality.

**Figure 3:**
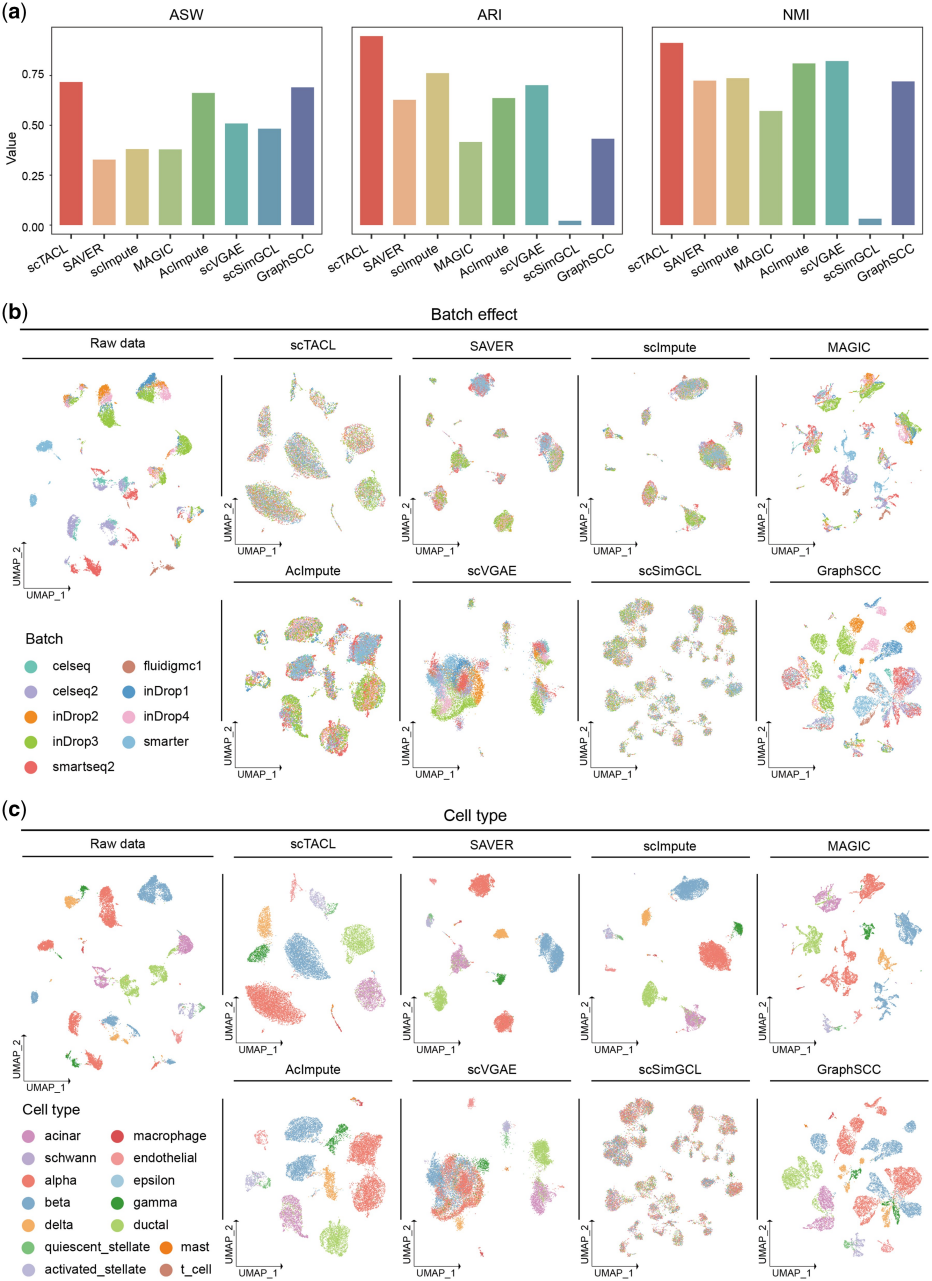
scTACL effectively removes batch effects in human pancreatic scRNA-seq datasets generated using different protocols. (a) Quantitative comparison of batch-effect correction performance across different methods on the pancreas dataset, assessed using cell-type average silhouette width (cell-type ASW), adjusted Rand index (ARI), and normalized mutual information (NMI). (b) UMAP visualization of cells colored by batch annotation after batch-effect correction. (c) UMAP visualization of cells colored by cell identity after batch-effect correction.

**Figure 4:**
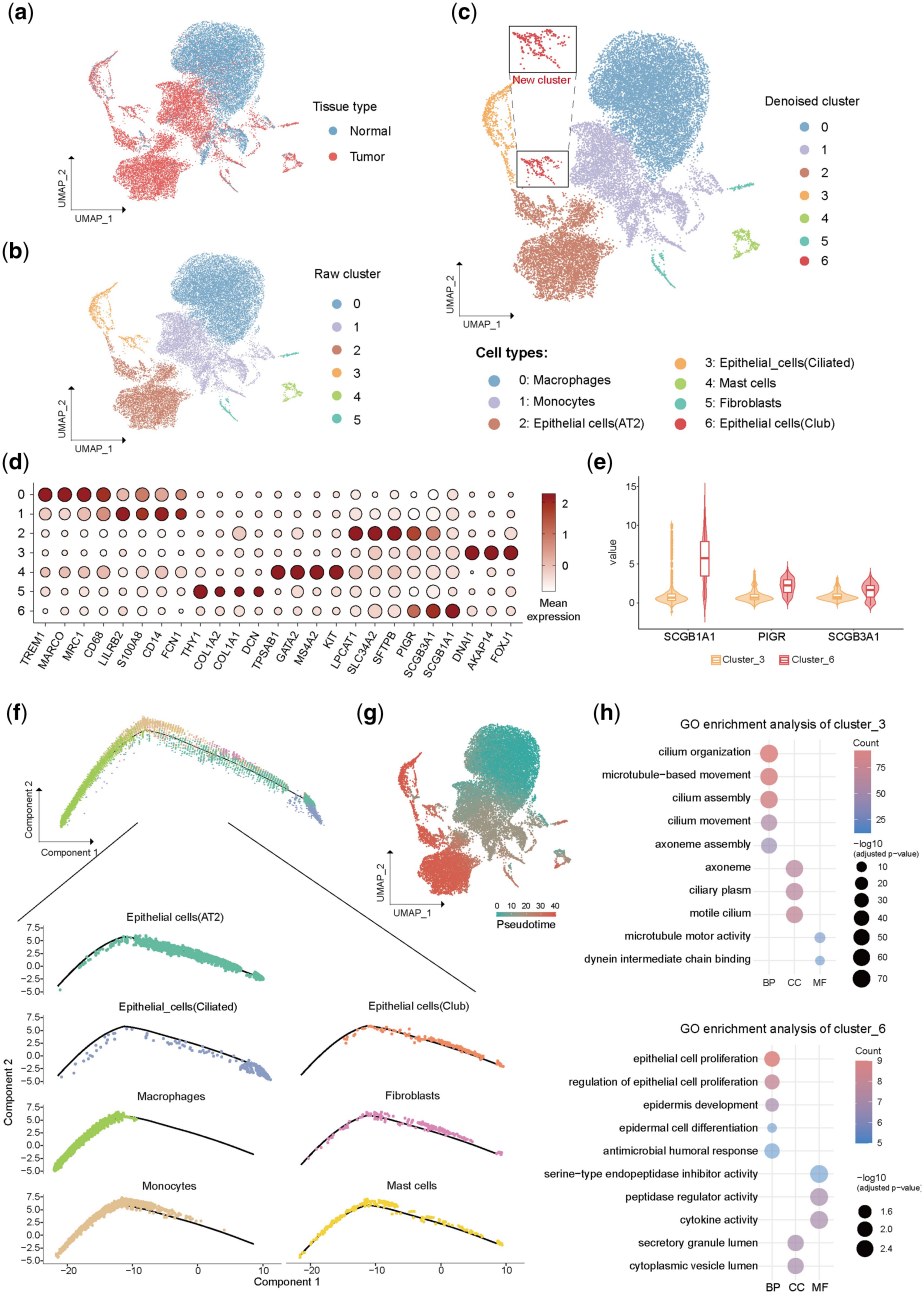
The experiment results on the LUAD datasets. (a) UMAP visualization of cells colored by tissue type. (b) UMAP visualization of cells colored by the raw clustering results. (c) UMAP visualization of the scTACL-denoised data, with cells colored according to the clusters identified after scTACL denoising. Newly identified clusters are highlighted with red circles. (d) Dot plot showing the expression of selected marker genes across cell clusters. (e) Violin plots showing the differential expression of selected genes between cluster 3 and cluster 6. (f) Pseudotime trajectory analysis of cells grouped by cell type. (g) UMAP visualization of pseudo-timing analysis results. (h) GO enrichment analysis of cluster 3 and cluster 6.

The results demonstrate that scTACL performed exceptionally well in this task, achieving the highest PCC values in 11 out of the 16 datasets and ranking within the top 3 for the remaining 5 datasets. On the Young dataset, scTACL achieved a PCC of 0.964, with the imputed scatter points distributed almost uniformly along the diagonal, showing excellent linear consistency (Fig. 2d). This finding indicates that scTACL not only effectively recovers expression values lost due to dropout events but also accurately preserves the original biological signals.

Importantly, a high PCC value does not mean that the imputation method simply copies the original data without changes. Instead, it demonstrates that the method effectively restores dropout events while maintaining consistency with the original expression patterns. This finding is supported by the better clustering performance of scTACL and subsequent experiments on batch effect removal. In summary, scTACL offers significant advantages in data imputation, achieving top results in correlation metrics while ensuring the accuracy and reliability of downstream analyses. Its high-quality imputation forms a strong base for later tasks, such as identifying cell subpopulations and removing batch effects. The overall performance of scTACL will be further emphasized in the following sections.

It is worth noting that the performance improvement of scTACL cannot be solely attributed to imputation. While imputing dropout events improves data completeness, the major gains arise from the joint learning of graph-based representations and topology-aware contrastive objectives. Importantly, scTACL does not arbitrarily inflate missing values. The recovered expression values are constrained by both neighborhood topology and the ZINB-based probabilistic model, which reduces the risk of introducing false-positive signals. Consistently, the imputation accuracy evaluation under different simulated dropout rates further supports this point (Additional file 6: Table 6, available as supplementary data at *Bioinformatics* online): scTACL achieves the lowest reconstruction errors across all masking levels (dropout = 0.10/0.30/0.50), with MSE of 0.11/0.15/0.15 and RMSE of 0.33/0.38/0.38, outperforming representative imputation baselines such as SAVER, scImpute, MAGIC, and AcImpute. This finding is further supported by the consistent improvements observed in clustering, batch effect removal, and trajectory inference tasks, which are highly sensitive to the recovery of spurious expression.

### 3.3 scTACL can effectively eliminate batch effects

Batch effects are systematic biases caused by non-biological factors in biological experiments, such as experiment timing, personnel, or sequencing platforms. These biases can significantly impact data consistency and lead to more errors in subsequent analyses. Furthermore, dropout events further diminish the accuracy of the results. Recent research indicates that enhancing data quality can significantly improve the effectiveness of batch effect removal methods, thereby effectively minimizing the influence of batch effects on analysis outcomes (Yuan *et al*. 2017, Luecken *et al*. 2022).

To achieve this, we utilized a publicly available pancreatic dataset comprising 16 382 cells and 19 093 genes from various sequencing platforms (e.g. CEL-seq (Hashimshony *et al*. 2012), CEL-seq2, Fluidigm C1 (Pollen *et al*. 2014), SMARTer, InDrop, and Smart-seq). First, we applied scTACL to perform data imputation on the original expression matrix. Then, we used the Harmony algorithm (Korsunsky *et al*. 2019) from the Scanpy package to correct batch effects in the imputed data. To verify scTACL’s effectiveness in removing batch effects, we established a control group that applied the same processing pipeline as other imputation algorithms. For the clustering algorithms mentioned earlier, we ensured the use of consistent analysis workflows for their embeddings to ensure comparable results. Figure 3a compares scTACL with various existing methods for removing batch effects. Figure 3a and Additional file 7: Table 7, available as supplementary data at *Bioinformatics* online show that scTACL achieved excellent performance on evaluation metrics such as ASW, ARI, and NMI. Notably, Table 7, available as supplementary data at *Bioinformatics* online also includes a comparison with Harmony, where scTACL consistently attains higher ASW, ARI, and NMI values. These results further highlight the strong capability of scTACL to enhance cell-type consistency across batches. Furthermore, Fig 3b and c present the UMAP embeddings of different methods for visual comparison. In addition, the UMAP embedding produced by Harmony is shown in Additional file 7: Fig. 5, available as supplementary data at *Bioinformatics* online for reference.

**Figure 5:**
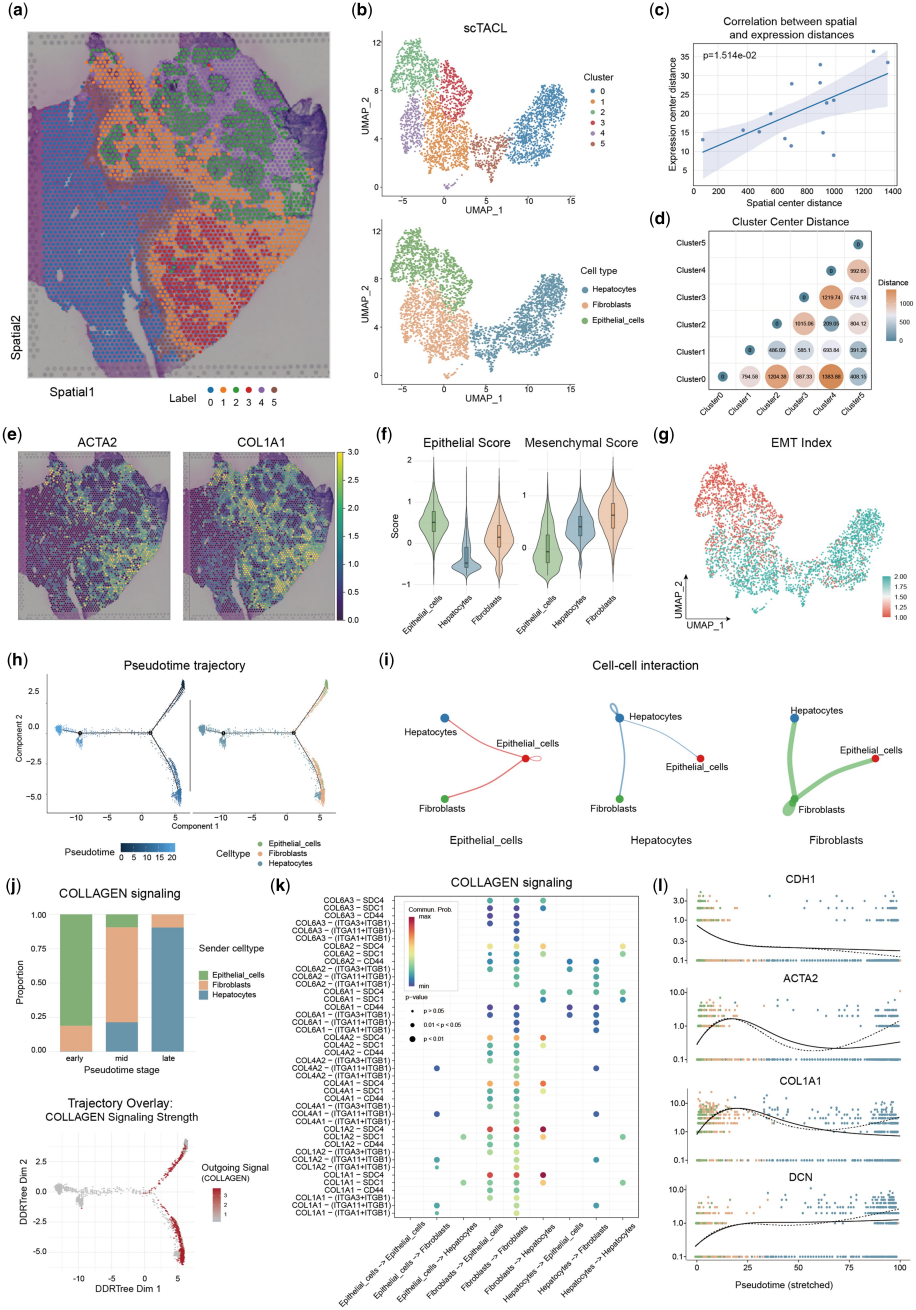
The experimental results of scTACL on the spatial transcriptome liver cancer dataset. (a) Cluster visualization results generated by scTACL. (b) UMAP visualization of cells colored by cluster assignment (top) and cell type annotation (bottom). (c) Correlation between spatial distance and gene expression distance. (d) Heatmap showing the relationships among cluster centers. (e) Visualization of marker fibroblast populations. (f) EMT status scores across three cell types. (g) UMAP visualization of cells colored by EMT score. (h) Pseudo-time trajectory analysis results. (i) Cell-cell interaction results among three cell types. (j) Proportion of cell types sending COLLAGEN signals across different pseudotime stages (upper panel). Pseudotime trajectory plots show the spatial distribution of COLLAGEN signal intensity, with higher intensity indicating stronger signals. (k) Correlation analysis of COLLAGEN signal in different cell types, where the size of the point represents the significance level of the *P*value. (l) Dynamic expression trends of EMT marker genes along pseudotime.

These figures show that although most methods reduce batch effects to varying degrees (except for MAGIC, scVGAE, and GraphSCC), some limitations still exist. For example, scSimGCL has a relatively loose structure with uneven color distributions, indicating poor consistency in clustering. Compared to other methods, SAVER, scImpute, and AcImpute demonstrate better batch effect removal; however, they still exhibit noticeable color stratification, indicating residual boundaries between batches. In contrast, scTACL has significant advantages: colors (representing different sequencing platforms) are highly mixed, and cluster structures are clearly defined. These findings indicate that scTACL effectively eliminates batch effects while preserving biologically meaningful cell type distributions. For instance, activated and quiescent stellate cell subtypes can be accurately distinguished and show clear clustering patterns, highlighting scTACL’s ability to retain subtype heterogeneity (Fig. 3c).

To further assess scalability, we benchmarked scTACL on a large-scale, multi-batch dataset comprising more than 70 000 cells, with random subsampling ranging from 10% to 70%. The results demonstrate favorable CPU/GPU memory scalability and runtime performance compared with baseline methods (Additional file 8: Table 8 and 9, available as supplementary data at *Bioinformatics* online).

In summary, scTACL not only outperforms in quantitative metrics but also matches its visualization results, further emphasizing its overall strengths in batch effect removal and data imputation.

### 3.4 scTACL can resolve differences between epithelial cell subtypes in LUAD data

To assess scTACL’s ability to identify fine-grained cellular subtypes, we applied it to a single-cell transcriptomic dataset from LUAD patients (Bischoff *et al*. 2021). This dataset comprises tumor and adjacent normal samples, consisting of 23 718 cells (12 845 tumor cells and 10 873 adjacent normal cells) and 26 294 genes (Fig. 4a). First, we clustered the raw data into six cell groups using the Leiden clustering algorithm (Fig. 4b). Following imputation of the original expression matrix with scTACL and reclustering under the same parameters, seven distinct cell clusters were identified (Fig. 4c). Notably, in the denoised clustering results, cells initially assigned to Cluster 3 were further divided into two separate clusters (Clusters 3 and 6), demonstrating that scTACL can reveal subtle cellular subtype differences that were not detectable in the raw data.

To further annotate cell types, we combined SingleR’s automatic annotations with manual curation based on known marker genes (Aran *et al*. 2019), categorizing all cells into five major groups: Macrophages, Monocytes, Epithelial cells, Mast cells, and Fibroblasts. The epithelial cells were further subdivided into three subtypes: AT2, ciliated, and club cells. Figure 4d shows the expression patterns of representative marker genes across cell clusters. The results revealed significant differences in marker gene expression between Cluster 3 and Cluster 6. Specifically, SCGB1A1, PIGR, and SCGB3A1, which are classical marker genes for club cells (Zhu *et al*. 2019, Zuo *et al*. 2020, Huang *et al*. 2023), were highly expressed in Cluster 6 but expressed at low levels in Cluster 3 (Fig. 4e). These findings further confirm that these two clusters represent two distinct epithelial cell subtypes: ciliated and club cells.

To investigate the functional changes in each cell subtype during LUAD progression, we performed pseudotime analysis using Monocle2, constructing the cell differentiation trajectory (Fig. 4f) and visualizing the pseudotime distribution with UMAP embedding (Fig. 4g). The analysis revealed that immune-related cells, including macrophages and monocytes, are present at the early stage of the differentiation trajectory, whereas mast cells and fibroblasts are distributed throughout the trajectory. In contrast, epithelial cells mostly appear in the middle and late stages of the trajectory. Among epithelial cells, AT2 cells are widely spread along the trajectory, whereas the two subtypes—club and ciliated cells—display distinct differentiation patterns: club cells are concentrated in the middle, while ciliated cells are mainly found at the endpoint of the trajectory. These results imply that different epithelial cell subtypes may serve distinct functional roles within the tumor microenvironment.

Further GO enrichment analysis highlighted functional differences between Cluster 3 and Cluster 6 (Fig. 4h). Cluster 3 was enriched in biological processes such as “cilium organization,” “cilium movement,” and “axoneme assembly,” which are closely linked to ciliary structure and motility, suggesting that these cells have typical motile cilia functions—a trait consistent with ciliated cell characteristics. Ciliated cells mainly function to clear foreign particles from the respiratory tract through ciliary motion, and their presence in LUAD tissues may imply that some tumor cells retain certain differentiated traits. Conversely, Cluster 6 was enriched in processes such as “epithelial cell proliferation,” “epidermis development,” “antimicrobial humoral response,” and “cytokine activity,” indicating strong proliferative and immunoregulatory capabilities—features characteristic of club cells. These nonciliated, secretory epithelial cells release various proteins involved in airway maintenance and immune responses. Prior studies suggest that club cells may serve as potential origin cells for KRAS-driven LUAD (Chen *et al*. 2022).

In summary, scTACL demonstrated an outstanding ability to resolve cellular heterogeneity in LUAD single-cell data, effectively distinguishing subtle subtype differences within epithelial cells. Notably, it excelled in identifying two functionally distinct epithelial subpopulations—club and ciliated cells. These findings emphasize scTACL as a powerful tool for uncovering cellular lineage relationships and the mechanisms of functional differentiation underlying LUAD progression.

### 3.5 scTACL clustering results show spatial structural consistency

To confirm the spatial consistency of the cell types identified by scTACL, we used a spatial transcriptomics dataset for liver cancer generated through the 10× Visium platform (Garbarino *et al*. 2023). First, without including any spatial position information, we performed clustering analysis on the dataset using scTACL, which resulted in six clusters (Fig. 5a and b). Using the SingleR tool and marker gene visualization, these clusters were annotated into three cell types (Fig. 5b): fibroblasts (Clusters 1 and 4), epithelial cells (Clusters 2 and 3), and hepatocytes (Clusters 0 and 5).

To evaluate whether the clusters identified by scTACL are consistent with the biological assumption of spatial structure consistency (i.e. cells with similar gene expression tend to be spatially proximal), we analyzed the relationship between spatial distance and gene expression distance among clusters. Specifically, for each cluster we defined a spatial center as the mean physical coordinates $\left(x,y\right)$ of all cells within the cluster, and an expression center as the mean vector of the PCA embeddings of its constituent cells. Pairwise Euclidean distances were then calculated between cluster centers to obtain spatial center distances and expression center distances.

As shown in Fig. 5c, spatial center distance is positively correlated with expression center distance (p = 1.514e-02), indicating that clusters that are farther apart in physical space tend to exhibit greater transcriptional differences. To further determine whether this relationship is an inherent property of the raw data or primarily introduced by the model, we repeated the same analysis using the raw count matrix. The results also show a significant positive correlation between spatial center distance and expression center distance (p = 2.98e-02; Additional file 9: Fig. 6, available as supplementary data at *Bioinformatics* online), suggesting that spatial–expression consistency is already present in the original data. Notably, the correlation obtained using scTACL exhibits stronger statistical significance, indicating that the representations learned by scTACL further preserve and enhance this biologically meaningful spatial–expression relationship.

**Figure 6:**
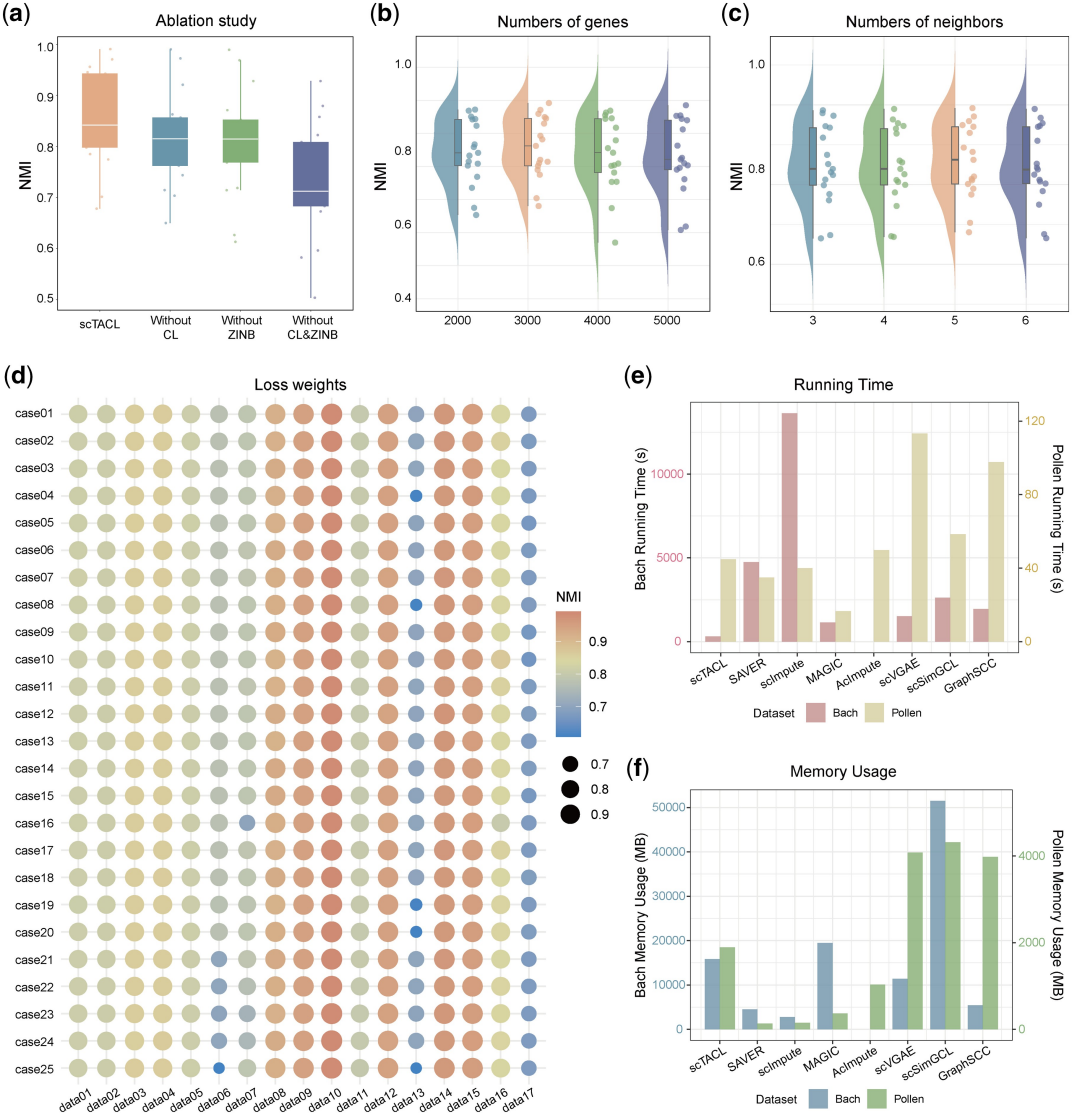
Robustness and efficiency analysis of scTACL. (a) Ablation study of topological domain-aware contrastive learning and ZINB distribution. (b) The NMI index was used to evaluate the clustering quality under conditions with different numbers of HVGs. (c) The NMI index was used to evaluate the clustering quality under conditions with different numbers of neighbors. (d) The NMI bubble plot under different loss weight assignments. (e) Comparison of scTACL and baseline methods in terms of running time. The left *y*-axis represents the running time on the Bach dataset, and the right *y*-axis represents the running time on the Pollen dataset. (f) Comparison of scTACL and baseline methods in terms of CPU usage. The left *y*-axis indicates CPU usage on the Bach dataset, and the right *y*-axis indicates CPU usage on the Pollen dataset.

In addition, the bubble chart in Fig. 5d visualizes the spatial distances between clusters, with bubble size and color representing distance, intuitively reflecting the relative distribution and proximity of clusters in spatial organization. This chart highlights the heterogeneity of clusters in the spatial dimension.

To further validate the biological plausibility of the scTACL clustering results, we systematically analyzed the EMT process, focusing on the dynamic changes in EMT-related genes across cell types and developmental stages. Figure 5e displays the spatial distribution of key EMT genes, ACTA2 and COL1A1, within the spatial transcriptomic slice. Both genes are highly expressed in fibroblast-enriched regions, indicating their central role in maintaining the mesenchymal phenotype. Additionally, using the EMTome database (Vasaikar *et al*. 2021), we constructed epithelial and mesenchymal feature scoring systems to evaluate the EMT state of the three cell types (Fig. 5f). The violin plot shows that epithelial cells have significantly higher epithelial scores, while fibroblasts have higher mesenchymal scores, consistent with EMT differentiation features. The EMT index visualized in Fig. 5g presents a transparent gradient in the UMAP space, revealing the dynamic distribution of EMT states across cell populations. These findings demonstrate that even without spatial position information, scTACL can accurately distinguish different cell types and effectively identify cellular phenotypic states.

EMT is a crucial biological process in tumor metastasis, tissue remodeling, and fibrosis, during which epithelial cells gradually lose their epithelial traits and acquire mesenchymal features, such as migratory and invasive abilities. Pseudotime trajectory analysis using Monocle2 (Fig. 5h) revealed that cells initially reside in an epithelial state and differentiate into two distinct lineages: fibroblasts and hepatocytes. This finding aligns with the EMT-induced differentiation pathway.

We further analyzed the cell–cell communication patterns among the three cell types (Fig. 5i). These results indicate that fibroblasts show significant communication activity with hepatocytes and epithelial cells, especially through the COLLAGEN signaling axis, which relays essential extracellular matrix remodeling information. Pseudotime trajectory analysis of the COLLAGEN signaling pathway revealed dynamic changes in the composition of sender cells at different time points (Fig. 5j). To clarify the signaling mechanism, we analyzed the communication probabilities of specific ligand–receptor pairs within the COLLAGEN pathway (Fig. 5k). The results showed that fibroblasts send high-intensity signals to other cell types via complexes such as COL1A1/COL1A2-ITGA1+ITGB1 (*P* < 0.01), which serve as key signaling hubs in the EMT process.

Finally, we analyzed the expression dynamics of EMT marker genes along the single-cell pseudotime trajectory (Fig. 5l). A typical EMT pattern was observed: The expression of the epithelial marker gene CDH1 gradually decreased along the trajectory, while the expression of the mesenchymal marker genes ACTA2, COL1A1, and DCN progressively increased, with COL1A1 and ACTA2 expression peaking in fibroblasts. This finding further supports the idea that as cells move along the pseudotime trajectory, epithelial cells lose their epithelial traits (CDH1) and gain mesenchymal traits (ACTA2, COL1A1).

In summary, scTACL achieves high-precision cell clustering without relying on spatial position information and uncovers cell–cell communication and gene dynamics during the EMT.

### 3.6 Robustness and efficiency analysis of scTACL

To assess the robustness of the scTACL model and the effectiveness of its components, we conducted a series of ablation experiments to analyze the specific contributions of its core modules (Fig. 6a, Additional file 10: Table 10, available as supplementary data at *Bioinformatics* online). The scTACL framework includes two main modules: a domain-aware contrastive learning module and a ZINB module. We tested clustering performance on 17 single-cell datasets by removing each module separately, and the results are as follows: Without the contrastive learning module (Without_CL), the model’s performance declined, with the average NMI dropping from 0.8511 to 0.8140 and the ARI decreasing from 0.8277 to 0.7351. Without the ZINB module (Without_ZINB), NMI decreased to 0.8075, and ARI dropped to 0.7328, showing a similar performance decline as when the contrastive learning module was removed. Without both modules (Without_CL&ZINB), NMI fell sharply to 0.7266, and ARI declined to 0.5918, marking the most significant performance drop and the worst overall model performance. In addition, removing the topological decoder (Without_TD) also leads to a further performance decline compared with the full scTACL model.

Taken together, these results demonstrate that each proposed component plays an important role in the overall framework. In particular, the degradation observed after removing the ZINB module further confirms the effectiveness of incorporating probabilistic modeling of gene expression counts, while the contrastive learning module enhances the structural representation of cells. Their joint integration enables scTACL to achieve improved clustering performance across diverse datasets.

Notably, on a small subset of datasets, removing a single module may yield marginal improvements, possibly due to reduced model complexity or dataset-specific characteristics. Nevertheless, the overall trend is consistent: the full scTACL framework benefits from the complementary effects of scTACL, ZINB-based probabilistic modeling, and topological decoding. As shown in Additional file 10: Table 10 and Fig. 7, available as supplementary data at *Bioinformatics* online, removing the contrastive learning module or the ZINB module alone causes only moderate relative decreases in average performance (NMI: 4.36%/5.12%; ARI: 11.19%/11.47%), whereas removing both results in substantial degradation (NMI: 14.63%; ARI: 28.50%). Moreover, removal of the topological decoder also leads to notable declines (NMI: 6.77%; ARI: 14.23%). Together, these findings highlight the synergistic contribution of the three components to the robust generalization ability of scTACL.

In this study, we systematically examined the impact of two key hyperparameters: the number of HVGs and the number of neighbors used to build the adjacency matrix. Based on previous literature (Inoue 2024, Zhang *et al*. 2024), we set reasonable parameter ranges, choosing HVG counts from [2000, 3000, 4000, 5000] and neighbor numbers from [3, 4, 5, 6]. To thoroughly assess the performance of scTACL, we performed extensive hyperparameter search experiments across all benchmark datasets. As shown in Fig. 6b and c, the results indicate that scTACL is insensitive to changes in these hyperparameters, demonstrating strong stability and robustness. Detailed results are available in Additional file 10: Tables 11 and 12, available as supplementary data at *Bioinformatics* online.

To assess how the weights of the loss function components affect model performance, we fix α=5 and conduct experiments with β,γ∈{0.3, 0.5, 0.6, 0.8, 1.0}, generating 25 combinations. As shown in Fig. 6d, the results reveal that for most datasets, NMI scores were largely insensitive to changes in β and γ. A few combinations exhibited minor fluctuations in specific datasets, but the overall performance remained consistent, further confirming the model’s robustness.

We also compared the runtime and memory usage of different models on two datasets: Bach (a large dataset with 23 184 cells and 19 965 genes) and Pollen (a small dataset with 301 cells and 21 721 genes) (Fig. 6e and f). scTACL achieved the shortest runtime on the Bach dataset and ranked fourth on the Pollen dataset, just behind SAVER, scImpute, and MAGIC. SAVER and scImpute used the least memory across both datasets, while scTACL showed moderate memory use, ranking fifth in both cases, demonstrating balanced performance. Notably, AcImpute was excluded from the Bach dataset comparison because its runtime exceeded 24 hours.

In summary, scTACL shows high accuracy, robustness, and efficiency. Its main modules significantly improve performance, and it stays stable across various hyperparameters and configurations. Additionally, scTACL utilizes resources efficiently and scales well, making it an ideal solution for large-scale single-cell datasets and demonstrating strong potential for widespread adoption.

## 4 Conclusion

This paper introduces a multitask scTACL framework that can be widely applied to single-cell transcriptomics data analysis, including core tasks such as data imputation, cell clustering, batch effect correction, and cell–cell interaction. The model combines a contrastive learning approach with ZINB modeling, enabling it to extract key features from raw data and fill in missing information effectively. Benchmark tests on 17 single-cell transcriptomics datasets from various sequencing platforms show that scTACL outperforms others in cell clustering and data imputation. On a pancreatic cancer dataset, scTACL significantly reduces batch effects caused by differences in technical platforms. In differential analysis with LUAD datasets, scTACL successfully identified two distinct epithelial cell subtypes and uncovered their potential biological differences through functional enrichment analysis. Additionally, without using spatial position information, scTACL accurately pinpointed epithelial and mesenchymal cell regions in spatial transcriptomics data from liver cancer, demonstrating its strong adaptability to spatial structures.

Through a series of robustness tests and parameter sensitivity analyses, we systematically evaluated the contributions of each module, the effects of parameter variations, and the model’s computational efficiency. The results indicate that scTACL maintains consistent performance across diverse settings, reflecting strong generalizability and practical potential. Nonetheless, several limitations remain. In addition to memory usage and scalability constraints, the current framework has not yet been fully validated on multi-modal datasets that integrate additional omics or imaging data, which may pose challenges for representation alignment. Moreover, potential biases introduced by the chosen graph construction strategy—e.g. the reliance on K-nearest neighbor graphs in PCA space—may affect model robustness when applied to datasets with uneven cell distributions or noisy feature spaces. Future work will address these aspects by exploring adaptive or data-driven graph learning mechanisms and extending the framework to multi-modal integration tasks.

Overall, scTACL is a robust, flexible, and comprehensive tool for single-cell transcriptomics analysis, providing unified support for various downstream tasks. Moreover, with the rapid advancement of single-cell transcriptomics technologies, most existing algorithms tend to focus on a single or partial task within the data analysis workflow. Although some generative methods based on large language models have accomplished end-to-end data analysis, their high computational resource requirements restrict their scalability in real-world applications. Therefore, we believe that a key focus for future research will be to create an integrated framework that combines data imputation, cell clustering, differential analysis, and other downstream tasks. This framework should strike a balance between comprehensive functionality and resource efficiency, paving the way for more practical and scalable solutions in single-cell transcriptomics analysis.

## Supplementary Material

btag361_Supplementary_Data

## Data Availability

The benchmarking datasets used in this study are available in https://figshare.com/s/e3b0ca6b8e3d4a619d5f. The source code for scTACL is available on GitHub at https://github.com/doriszmr/scTACL.

## References

[btag361-B1] Aran D , Looney AP, Liu L et al Reference-based analysis of lung single-cell sequencing reveals a transitional profibrotic macrophage. Nat Immunol 2019;20:163–72. 30643263 10.1038/s41590-018-0276-y PMC6340744

[btag361-B2] Bischoff P , Trinks A, Obermayer B et al Single-cell RNA sequencing reveals distinct tumor microenvironmental patterns in lung adenocarcinoma. Oncogene 2021;40:6748–58. 34663877 10.1038/s41388-021-02054-3 PMC8677623

[btag361-B3] Chen Y , Toth R, Chocarro S et al Club cells employ regeneration mechanisms during lung tumorigenesis. Nat Commun 2022;13:4557. 35931677 10.1038/s41467-022-32052-2 PMC9356049

[btag361-B4] Garbarino O , Lambroia L, Basso G et al Spatial resolution of cellular senescence dynamics in human colorectal liver metastasis. Aging Cell 2023;22:e13853. 37157887 10.1111/acel.13853 PMC10352575

[btag361-B5] Han Y , Wang Y, Dong X et al TISCH2: expanded datasets and new tools for single-cell transcriptome analyses of the tumor microenvironment. Nucleic Acids Res 2023;51:D1425–D1431. 36321662 10.1093/nar/gkac959 PMC9825603

[btag361-B6] Hashimshony T , Senderovich N, Avital G et al CEL-Seq2: sensitive highly-multiplexed single-cell RNA-Seq. Genome Biol 2016;17:1–7. 27121950 10.1186/s13059-016-0938-8 PMC4848782

[btag361-B7] Hashimshony T , Wagner F, Sher N et al CEL-Seq: single-cell RNA-Seq by multiplexed linear amplification. Cell Rep 2012;2:666–73. 22939981 10.1016/j.celrep.2012.08.003

[btag361-B8] Huang FW , Song H, Weinstein HNW et al Club‐like cells in proliferative inflammatory atrophy of the prostate. J Pathol 2023;261:85–95. 37550827 10.1002/path.6149 PMC10527202

[btag361-B9] Huang M , Wang J, Torre E et al SAVER: gene expression recovery for single-cell RNA sequencing. Nat Methods 2018;15:539–42. 29941873 10.1038/s41592-018-0033-z PMC6030502

[btag361-B10] Inoue Y. Scvgae: a novel approach using zinb-based variational graph autoencoder for single-cell RNA-seq imputation. arXiv preprint arXiv: 2403.08959, 2024, preprint: not peer reviewed.

[btag361-B11] Kingma DP , Welling M. Auto-Encoding Variational Bayes. In: *Proceedings of the 2nd International Conference on Learning Representations (ICLR 2014)*. Banff, Canada: OpenReview.net; 2014.

[btag361-B12] Kipf TN , Welling M. Semi-supervised classification with graph convolutional networks. In: *Proceedings of the International Conference on Learning Representations (ICLR 2017)*. Toulon, France: OpenReview.net; 2017.

[btag361-B13] Klein AM , Mazutis L, Akartuna I et al Droplet barcoding for single-cell transcriptomics applied to embryonic stem cells. Cell 2015;161:1187–201. 26000487 10.1016/j.cell.2015.04.044 PMC4441768

[btag361-B14] Korsunsky I , Millard N, Fan J et al Fast, sensitive and accurate integration of single-cell data with harmony. Nat Methods 2019;16:1289–96. 31740819 10.1038/s41592-019-0619-0 PMC6884693

[btag361-B15] Li WV , Li JJ. An accurate and robust imputation method scImpute for single-cell RNA-seq data. Nat Commun 2018;9:997. 29520097 10.1038/s41467-018-03405-7 PMC5843666

[btag361-B16] Luecken MD , Buttner M, Chaichoompu K et al Benchmarking atlas-level data integration in single-cell genomics. Nat Methods 2022;19:41–50. 34949812 10.1038/s41592-021-01336-8 PMC8748196

[btag361-B17] Macosko EZ , Basu A, Satija R et al Highly parallel genome-wide expression profiling of individual cells using nanoliter droplets. Cell 2015;161:1202–14. 26000488 10.1016/j.cell.2015.05.002 PMC4481139

[btag361-B18] Mortazavi A , Williams BA, McCue K et al Mapping and quantifying mammalian transcriptomes by RNA-Seq. Nat Methods 2008;5:621–8. 18516045 10.1038/nmeth.1226 PMC13303166

[btag361-B19] Picelli S , Bjoeklund AK, Faridani OR et al Smart-seq2 for sensitive full-length transcriptome profiling in single cells. Nat Methods 2013;10:1096–8. 24056875 10.1038/nmeth.2639

[btag361-B20] Pollen AA , Nowakowski T, Shuga J et al Low-coverage single-cell mRNA sequencing reveals cellular heterogeneity and activated signaling pathways in developing cerebral cortex. Nat Biotechnol 2014;32:1053–8. 25086649 10.1038/nbt.2967 PMC4191988

[btag361-B21] Ramsköld D , Luo S, Wang Y-C et al Full-length mRNA-Seq from single-cell levels of RNA and individual circulating tumor cells. Nat Biotechnol 2012;30:777–82. 22820318 10.1038/nbt.2282 PMC3467340

[btag361-B22] Stuart T , Butler A, Hoffman P et al Comprehensive integration of single-cell data. Cell 2019;177:P1888–902.E1821. 31178118 10.1016/j.cell.2019.05.031 PMC6687398

[btag361-B23] Tang F , Barbacioru C, Wang Y et al mRNA-Seq whole-transcriptome analysis of a single cell. Nat Methods 2009;6:377–82. 19349980 10.1038/nmeth.1315

[btag361-B24] Van Dijk D , Sharma R, Nainys J et al Recovering gene interactions from single-cell data using data diffusion. Cell 2018;174:P716-29.E727. 29961576 10.1016/j.cell.2018.05.061 PMC6771278

[btag361-B25] Vasaikar SV , Deshmukh AP, den Hollander P et al EMTome: a resource for pan-cancer analysis of epithelial-mesenchymal transition genes and signatures. Br J Cancer 2021;124:259–69. 33299129 10.1038/s41416-020-01178-9 PMC7782839

[btag361-B26] Vo LT , Ha QT et al scZiva: imputation method for single-cell RNA-seq data with zero-inflated variational autoencoder. BMC Bioinformatics 2026;27:92. 41857511 10.1186/s12859-026-06422-2 PMC13122936

[btag361-B27] Wang S , Liu Y, Zhang H et al scE2EGAE: enhancing single-cell RNA-Seq data analysis through an end-to-end cell-graph-learnable graph autoencoder with differentiable edge sampling. Biol Direct 2025;20:66. 40426257 10.1186/s13062-025-00616-z PMC12108024

[btag361-B28] Wolf FA , Angerer P, Theis FJ. SCANPY: large-scale single-cell gene expression data analysis. Genome Biol 2018;19:1–5. 29409532 10.1186/s13059-017-1382-0 PMC5802054

[btag361-B29] Yao J , Li L, Xu T et al ZMGA: a ZINB-based multi-modal graph autoencoder enhancing topological consistency in single-cell clustering. Biomed Signal Process Control 2024;97:106587.

[btag361-B30] Yuan G-C , Cai L, Elowitz M et al Challenges and emerging directions in single-cell analysis. Genome Biol 2017;18:1–8. 28482897 10.1186/s13059-017-1218-y PMC5421338

[btag361-B31] Zeng Y , Zhou X, Rao J et al Accurately clustering single-cell RNA-seq data by capturing structural relations between cells through graph convolutional network. In: *2020 IEEE International Conference on Bioinformatics and Biomedicine (BIBM)*. pp. 519-522. IEEE, 2020.

[btag361-B32] Zhang W , Liu T, Zhang H et al AcImpute: a constraint-enhancing smooth-based approach for imputing single-cell RNA sequencing data. Bioinformatics 2025;41:btae711. 40037523 10.1093/bioinformatics/btae711 PMC11890269

[btag361-B33] Zhang Z , Liu Y, Xiao M et al Graph contrastive learning as a versatile foundation for advanced scRNA-seq data analysis. Brief Bioinform 2024;25:bbae558. 39487083 10.1093/bib/bbae558 PMC11530284

[btag361-B34] Zheng GX , Terry JM, Belgrader P et al Massively parallel digital transcriptional profiling of single cells. Nat Commun 2017;8:14049. 28091601 10.1038/ncomms14049 PMC5241818

[btag361-B35] Zhu L , An L, Ran D et al The club cell marker SCGB1A1 downstream of FOXA2 is reduced in asthma. Am J Respir Cell Mol Biol 2019;60:695–704. 30576223 10.1165/rcmb.2018-0199OC PMC6543749

[btag361-B36] Zuo W-L , Rostami MR, LeBlanc M et al Dysregulation of club cell biology in idiopathic pulmonary fibrosis. PLoS One 2020;15:e0237529. 32941426 10.1371/journal.pone.0237529 PMC7498242

