# Supplementary material for: scTACL: a multitask topology-aware contrastive learning approach for single-cell transcriptomics analysis
Source: Bioinformatics. 2026 Jun 4;42(6):btag361. doi: 10.1093/bioinformatics/btag361 (PMC13290474; doi:10.1093/bioinformatics/btag361)
Supplement: btag361_Supplementary_Data [file btag361_supplementary_data.docx]

**Supplementary file for scTACL: A multitask topology-aware contrastive learning approach for single-cell transcriptomics analysis**

**Additional file 1: Hyperparameters and Preprocessing Settings Used in scTACL**

Table S1. Key hyperparameters and preprocessing settings used in scTACL

| **Category** | **Hyperparameter** | **Description** | **Value** |
| --- | --- | --- | --- |
| Preprocessing | HVG selection method | Method for highly variable gene selection | seurat_v3 (Scanpy) |
|  | Number of HVGs | Number of selected highly variable genes | 3,000 |
|  | Batch correction | Batch integration method | Harmony |
|  | Harmony theta | Diversity clustering penalty parameter | 2 |
|  | Harmony lambda | Ridge regression penalty | 1 |
|  | Harmony max iterations | Maximum number of Harmony iterations | 20 |
| Graph construction | K (neighbors) | Number of nearest neighbors in KNN graph | 5 |
|  | Distance metric | Metric used for KNN construction | Euclidean (PCA space) |
| Model architecture | Embedding dimension | Dimension of latent representations | 96 |
|  | Number of GNN layers | Graph convolution layers | 1 |
| Training | Optimizer | Optimization algorithm | Adam |
|  | Learning rate | Initial learning rate | 8e-4 |
|  | Training epochs | Number of training epochs | 500 |
|  | Batch size | Mini-batch size | Full graph |
| Loss weights | $\alpha$ | Reconstruction loss weight | 5.0 |
|  | $\beta$ | Topological consistency loss weight | 0.6 |
|  | $\gamma$ | Contrastive loss weight | 0.6 |
| Augmentation | Feature perturbation | Node feature augmentation strategy | Random shuffling |
| Hardware | GPU | GPU used for training | NVIDIA Tesla A100 |

**Additional file 2: Summary of Benchmarking Methods and Datasets**

Table S2. Information of benchmark methods

| **NO.** | **Method** | **Platform** | **Task** | **Github** |
| --- | --- | --- | --- | --- |
| 1 | SAVER | R | Impute | https://github.com/mohuangx/SAVER |
| 2 | scImpute | R | Impute | https://github.com/Vivianstats/scImpute |
| 3 | MAGIC | Python | Impute | https://github.com/KrishnaswamyLab/MAGIC |
| 4 | AcImpute | Python | Impute | https://github.com/Liutto/AcImpute |
| 5 | scVGAE | Python | Cluster | https://github.com/inoue0426/scVGAE |
| 6 | scSimGCL | Python | Cluster | https://github.com/zhangzh1328/scSimGCL |
| 7 | GraphSCC | Python | Cluster | https://github.com/biomed-AI/GraphSCC |

Table S3. Information of benchmarking datasets

| **NO.** | **Dataset** | **Platform** | **Cells** | **Genes** | **Subtypes** | **Source** |
| --- | --- | --- | --- | --- | --- | --- |
| 1 | Adam | Drop-seq | 3660 | 23797 | 8 | Adam et al.,2017 |
| 2 | Bach | 10X | 23184 | 19965 | 8 | Bach et al.,2017 |
| 3 | Klein | inDrop | 2717 | 24047 | 4 | klein et al.,2015 |
| 4 | Muraro | CEL-seq2 | 2126 | 19127 | 10 | Muraro et al.,2016 |
| 5 | Plasschaert | inDrop | 6977 | 28205 | 8 | Plasschaert et al.,2018 |
| 6 | Tosches turtle | Drop-seq | 18664 | 23500 | 15 | Tosches et al.,2018 |
| 7 | Young | 10X | 5685 | 33658 | 11 | Young et al.,2018 |
| 8 | Pollen | SMARTer | 301 | 21721 | 11 | Pollen et al.,2014 |
| 9 | Quake 10x Bladder | 10X | 2432 | 22966 | 2 | Consortium,2020 |
| 10 | Quake 10x Limb Muscle | 10X | 3855 | 22966 | 6 | Consortium,2020 |
| 11 | Quake 10x Spleen | 10X | 9552 | 23341 | 5 | Schaum et al.,2018 |
| 12 | Quake Smart-seq2 Heart | Smart-seq2 | 4365 | 23341 | 8 | Schaum et al.,2018 |
| 13 | Quake Smart-seq2 Trachea | Smart-seq2 | 1350 | 23341 | 4 | Schaum et al.,2018 |
| 14 | Quake Smart-seq2 Diaphragm | Smart-seq2 | 870 | 23341 | 5 | Schaum et al.,2018 |
| 15 | Quake Smart-seq2 Limb Muscle | Smart-seq2 | 1090 | 23341 | 6 | Schaum et al.,2018 |
| 16 | Quake Smart-seq2 Lung | Smart-seq2 | 1676 | 23341 | 11 | Schaum et al.,2018 |
| 17 | Romanov | SMARTer | 2881 | 21143 | 7 | Romanov et al.,2017 |

**Additional file 3: Clustering Results on Benchmark Datasets**

Table S4. NMI results of benchmarking datasets

| **Dataset** | **scTACL** | **SAVER** | **scImpute** | **MAGIC** | **AcImpute** | **scVGAE** | **scSimGCL** | **GraphSCC** | **K-means** |
| --- | --- | --- | --- | --- | --- | --- | --- | --- | --- |
| Adam | 0.8116 | 0.7752 | 0.7192 | 0.7562 | 0.728 | 0.8538 | 0.8091 | 0.8874 | 0.7798 |
| Bach | 0.8099 | 0.7726 | 0.8204 | 0.7950 | NA | 0.8243 | 0.8136 | 0.8459 | 0.8214 |
| Klein | 0.8599 | 0.8041 | 0.7927 | 0.4397 | 0.6726 | 0.8621 | 0.8356 | 0.7874 | 0.7801 |
| Muraro | 0.8658 | 0.7435 | 0.8148 | 0.7635 | 0.8934 | 0.8925 | 0.8793 | 0.8391 | 0.8555 |
| Plasschaert | 0.8107 | 0.5007 | 0.5027 | 0.7452 | 0.0024 | 0.7309 | 0.7243 | 0.8500 | 0.8151 |
| Tosches_turtle | 0.7745 | 0.6699 | 0.6567 | 0.7090 | 0.7404 | 0.7200 | 0.7874 | 0.7640 | 0.7596 |
| Young | 0.7850 | 0.6729 | 0.6076 | 0.6994 | 0.7146 | 0.7339 | 0.7384 | 0.6532 | 0.6432 |
| Pollen | 0.9268 | 0.7930 | 0.7676 | 0.8581 | 0.9453 | 0.9385 | 0.8740 | 0.9284 | 0.9117 |
| Quake_10x_Bladder | 0.9453 | 0.5898 | 0.7281 | 0.6116 | 0.8100 | 0.7833 | 0.7877 | 0.8164 | 0.9453 |
| Quake_10x_Limb_Muscle | 0.9906 | 0.6618 | 0.6679 | 0.6671 | 0.8706 | 0.9891 | 0.8562 | 0.9531 | 0.9717 |
| Quake_10x_Spleen | 0.7978 | 0.5110 | 0.7073 | 0.7294 | 0.8364 | 0.6199 | 0.8538 | 0.7350 | 0.8461 |
| Quake_Smart-seq2_Heart | 0.9425 | 0.6756 | 0.8239 | 0.7062 | 0.8609 | 0.8323 | 0.7699 | 0.8316 | 0.9479 |
| Quake_Smart-seq2_Trachea | 0.7014 | 0.4981 | 0.6244 | 0.5992 | 0.8382 | 0.6242 | 0.5399 | 0.7080 | 0.7234 |
| Quake_Smart-seq2_Diaphragm | 0.9705 | 0.8269 | 0.6827 | 0.8049 | 0.8382 | 0.9637 | 0.9207 | 0.9738 | 0.9569 |
| Quake_Smart-seq2_Limb_Muscle | 0.9559 | 0.7626 | 0.8176 | 0.6744 | 0.8472 | 0.9430 | 0.8851 | 0.8847 | 0.9536 |
| Quake_Smart-seq2_Lung | 0.8418 | 0.6708 | 0.6497 | 0.7029 | 0.7548 | 0.8185 | 0.7438 | 0.8566 | 0.8376 |
| Romanov | 0.6781 | 0.5488 | 0.5350 | 0.6003 | 0.7206 | 0.6487 | 0.6393 | 0.6448 | 0.6745 |
| Mean | 0.8511 | 0.6751 | 0.7011 | 0.6978 | 0.7546 | 0.8105 | 0.7917 | 0.8211 | 0.8367 |
| Median | 0.8418 | 0.6729 | 0.7073 | 0.7062 | 0.8100 | 0.8243 | 0.8091 | 0.8391 | 0.8376 |

Table S5. ARI results of benchmarking datasets

| **Dataset** | **scTACL** | **SAVER** | **scImpute** | **MAGIC** | **AcImpute** | **scVGAE** | **scSimGCL** | **GraphSCC** | **K-means** |
| --- | --- | --- | --- | --- | --- | --- | --- | --- | --- |
| Adam | 0.7233 | 0.7157 | 0.6432 | 0.6864 | 0.6197 | 0.8377 | 0.7179 | 0.9028 | 0.6751 |
| Bach | 0.8016 | 0.8011 | 0.8433 | 0.8309 | NA | 0.8162 | 0.8080 | 0.8523 | 0.8156 |
| Klein | 0.8469 | 0.7525 | 0.7714 | 0.2939 | 0.6368 | 0.8497 | 0.7900 | 0.7803 | 0.7689 |
| Muraro | 0.9008 | 0.8061 | 0.8721 | 0.8256 | 0.9305 | 0.9340 | 0.9173 | 0.8814 | 0.9000 |
| Plasschaert | 0.8035 | 0.4448 | 0.4562 | 0.8088 | 0 | 0.6035 | 0.6061 | 0.8642 | 0.8020 |
| Tosches_turtle | 0.5615 | 0.4343 | 0.4313 | 0.4647 | 0.5012 | 0.5151 | 0.5859 | 0.6521 | 0.7521 |
| Young | 0.6736 | 0.5926 | 0.4720 | 0.5683 | 0.5549 | 0.6158 | 0.6675 | 0.4601 | 0.4583 |
| Pollen | 0.9133 | 0.6510 | 0.6519 | 0.8140 | 0.9493 | 0.9385 | 0.7810 | 0.9177 | 0.8953 |
| Quake_10x_Bladder | 0.9737 | 0.6196 | 0.7889 | 0.6526 | 0.7653 | 0.7385 | 0.7448 | 0.7588 | 0.9737 |
| Quake_10x_Limb_Muscle | 0.9953 | 0.4785 | 0.4836 | 0.5126 | 0.8116 | 0.9947 | 0.7912 | 0.9601 | 0.9873 |
| Quake_10x_Spleen | 0.8203 | 0.4741 | 0.8731 | 0.8925 | 0.9129 | 0.4886 | 0.9293 | 0.7782 | 0.9063 |
| Quake_Smart-seq2_Heart | 0.9692 | 0.5747 | 0.8818 | 0.6246 | 0.8814 | 0.6727 | 0.6114 | 0.7075 | 0.9726 |
| Quake_Smart-seq2_Trachea | 0.5836 | 0.2948 | 0.5713 | 0.5267 | 0.8429 | 0.5226 | 0.5510 | 0.5577 | 0.5613 |
| Quake_Smart-seq2_Diaphragm | 0.9868 | 0.8596 | 0.6985 | 0.8723 | 0.8429 | 0.9783 | 0.9524 | 0.9870 | 0.9726 |
| Quake_Smart-seq2_Limb_Muscle | 0.9734 | 0.6584 | 0.8203 | 0.5677 | 0.8189 | 0.9689 | 0.8780 | 0.9083 | 0.9684 |
| Quake_Smart-seq2_Lung | 0.8378 | 0.5924 | 0.5603 | 0.6552 | 0.5993 | 0.7408 | 0.5453 | 0.8472 | 0.8232 |
| Romanov | 0.7063 | 0.4906 | 0.5064 | 0.5629 | 0.7597 | 0.6208 | 0.6225 | 0.6145 | 0.7005 |
| Mean | 0.8277 | 0.6024 | 0.6662 | 0.6564 | 0.7142 | 0.7551 | 0.7353 | 0.7900 | 0.8196 |
| Median | 0.8378 | 0.5926 | 0.6519 | 0.6526 | 0.7653 | 0.7408 | 0.7448 | 0.8472 | 0.8232 |

**Additional file 4: UMAP Results of scTACL on Benchmark Datasets**


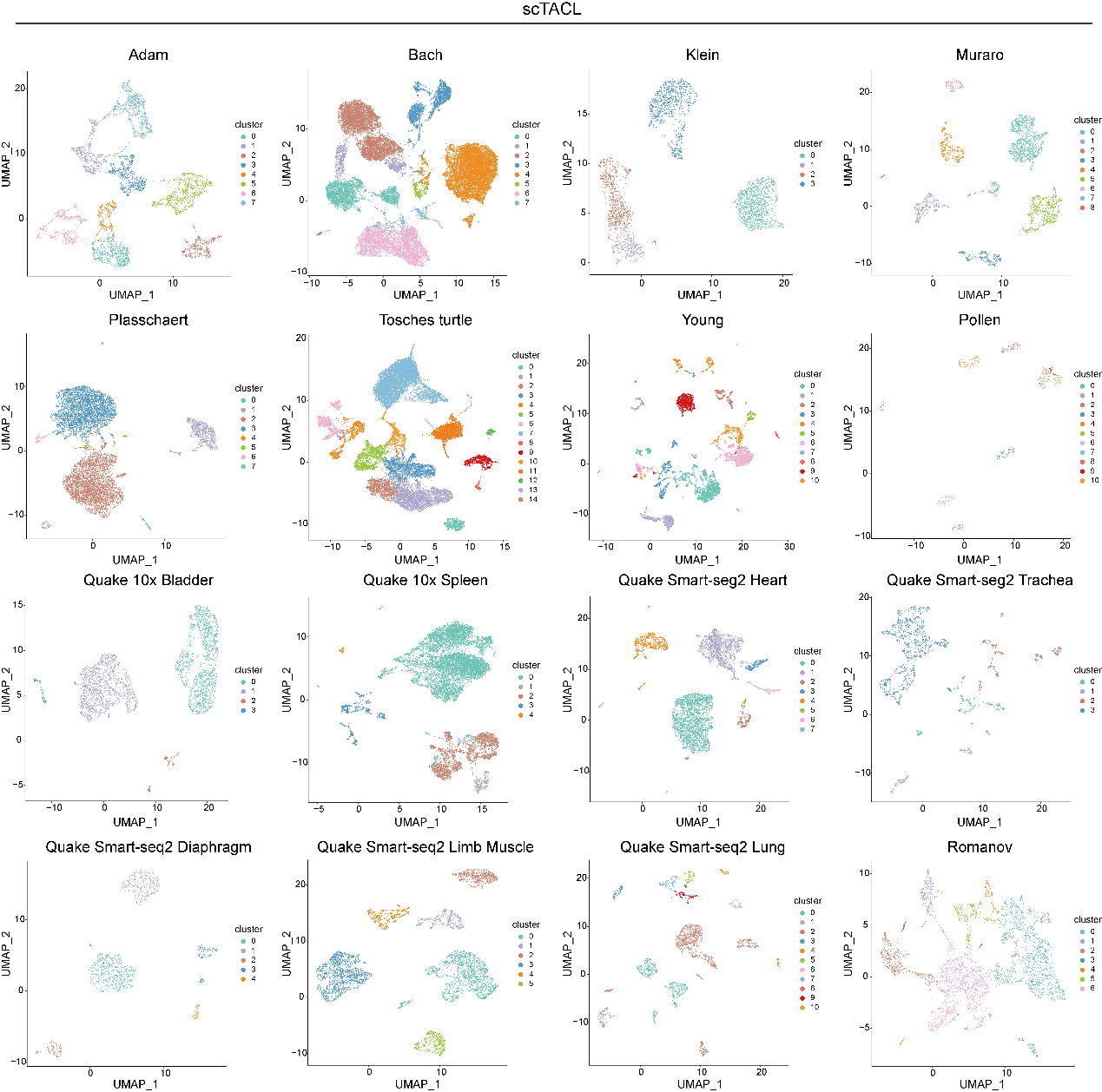


Figure S1. Clustering results of scTACL on 16 datasets, visualized using UMAP, with data points color-coded according to the clustering results.

**Additional file 5: Data Imputation of scTACL on Benchmark Datasets**


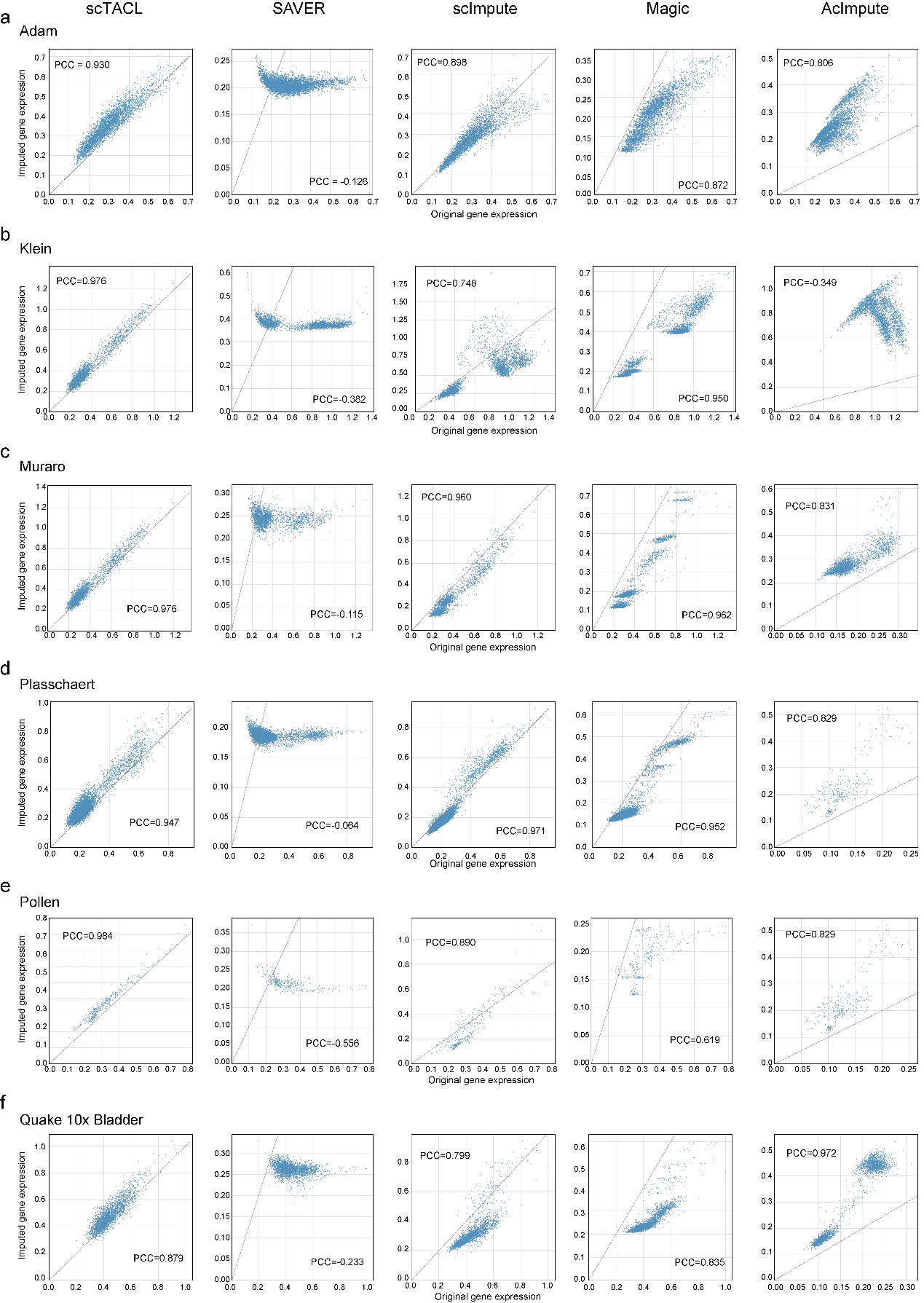
Figure S2. Comparison of data imputation performance between scTACL and baseline methods on the remaining 6 datasets.


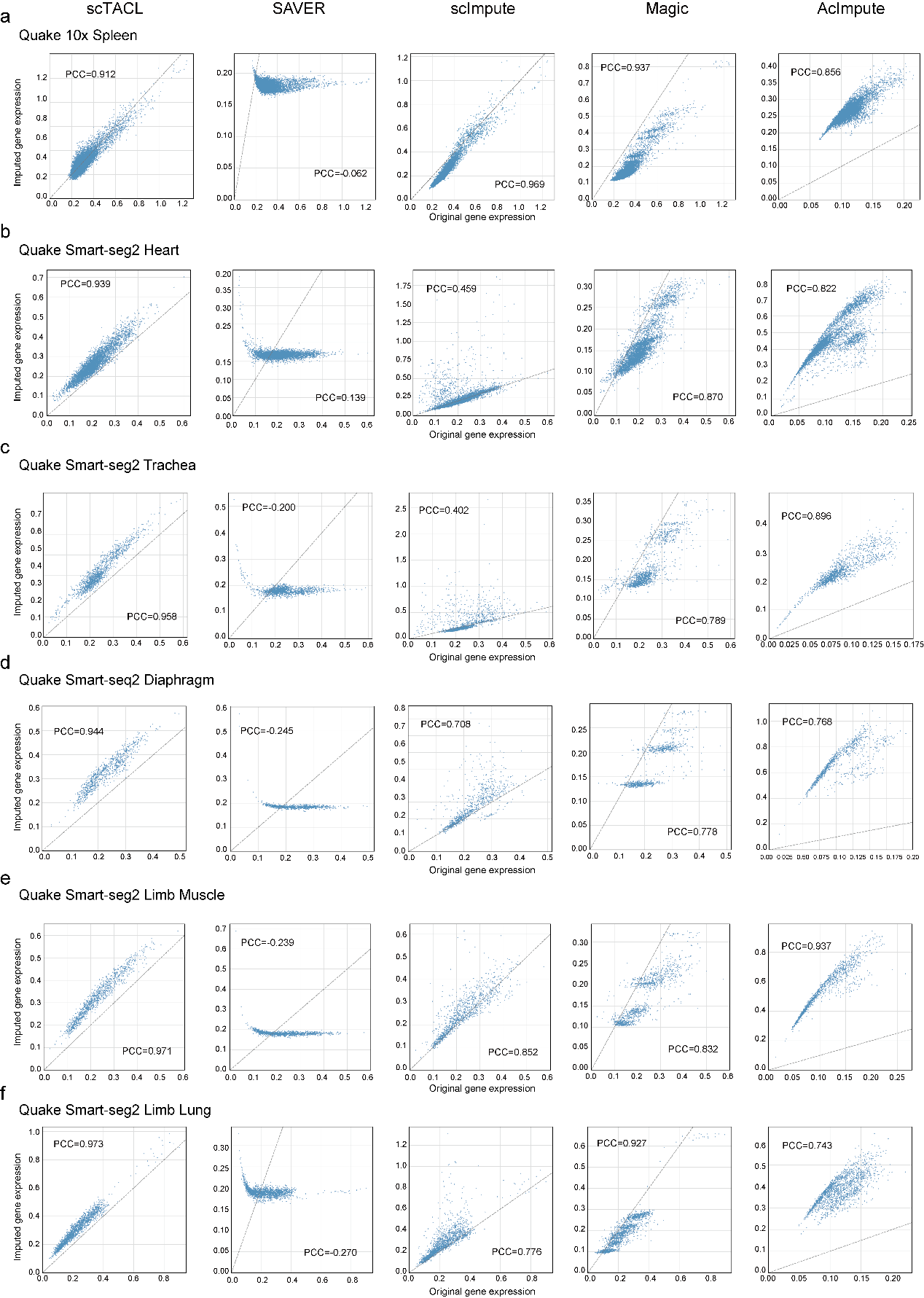


Figure S3. Comparison of data imputation performance between scTACL and baseline methods on the remaining 6 datasets.


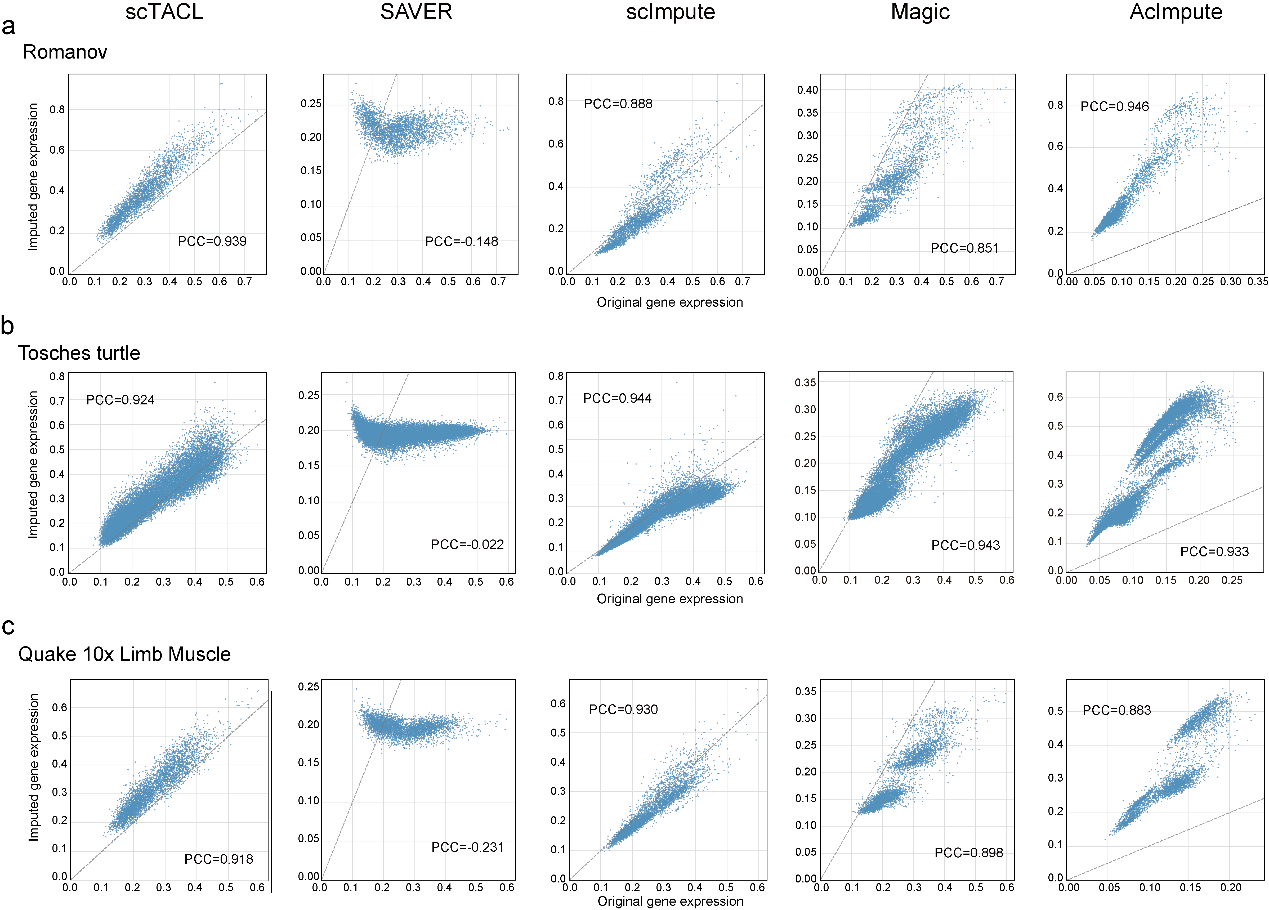
Figure S4. Comparison of data imputation performance between scTACL and baseline methods on the remaining 3 datasets.

**Additional file 6: Average Imputation Accuracy Under Different Dropout Rates**

Table S6. Average imputation accuracy (MSE/RMSE) under different dropout rates

| **Method** | **MSE (0.10)** | **RMSE (0.10)** | **MSE (0.30)** | **RMSE (0.30)** | **MSE (0.50)** | **RMSE (0.50)** |
| --- | --- | --- | --- | --- | --- | --- |
| scTACL | 0.11 | 0.33 | 0.15 | 0.38 | 0.15 | 0.38 |
| SAVER | 0.21 | 0.45 | 0.3 | 0.55 | 0.41 | 0.64 |
| scImpute | 0.58 | 0.73 | 0.7 | 0.8 | 1 | 0.91 |
| MAGIC | 0.5 | 0.7 | 0.53 | 0.73 | 0.57 | 0.75 |
| AcImpute | 0.32 | 0.53 | 0.26 | 0.48 | 0.18 | 0.41 |

**Additional file 7: Mean ASW, ARI, and NMI Scores of Batch Correction Methods**

Table S7. Mean ASW, ARI, and NMI scores of batch correction methods

| **Method** | **ASW** | **ARI** | **NMI** |
| --- | --- | --- | --- |
| scTACL | 0.7171 | 0.9468 | 0.9132 |
| Harmony | 0.6833 | 0.9268 | 0.8874 |
| SAVER | 0.3280 | 0.6277 | 0.7241 |
| scImpute | 0.3808 | 0.7612 | 0.7362 |
| MAGIC | 0.3791 | 0.4163 | 0.5719 |
| AcImpute | 0.6623 | 0.6363 | 0.8105 |
| scVGAE | 0.5091 | 0.7014 | 0.8223 |
| scSimGCL | 0.4831 | 0.0213 | 0.0316 |
| GraphSCC | 0.6903 | 0.4324 | 0.7197 |

**
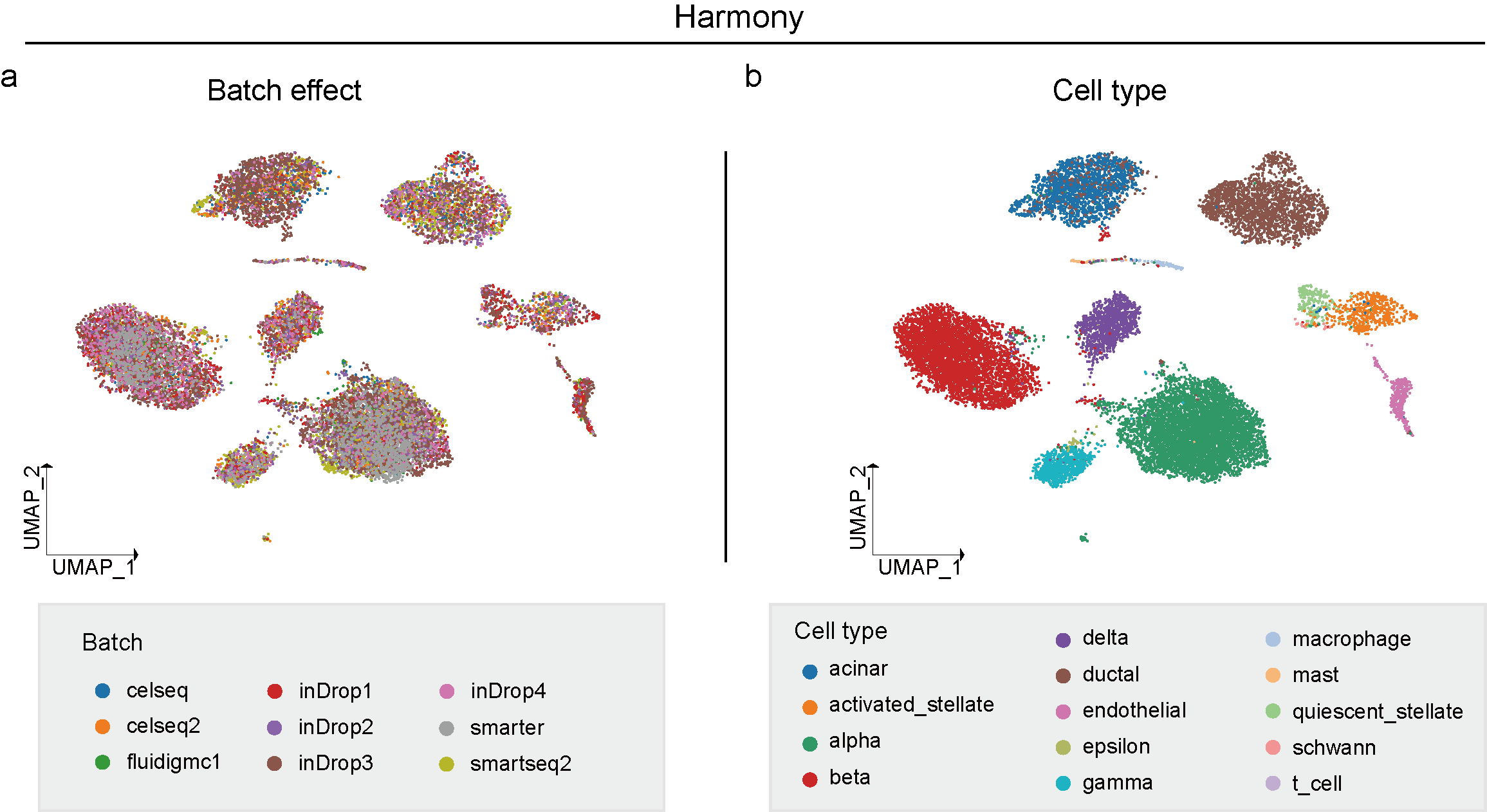
**

Figure S5. Batch effect correction performance of Harmony on human pancreatic data generated from different scRNA-seq protocols. (a) UMAP visualization, with data points colored by batch annotation. (b) UMAP visualization, with data points colored by cell type.

**Additional file 8: CPU/GPU Resource Consumption and Runtime under Different Subsampling Fractions on a Large-Scale Multi-Batch Dataset**

Table S8. CPU and GPU Resource Consumption under Different Fractions on Large-Scale Multi-Batch Datasets (MB)

| **Batch** | **Number of Cells** | **CPU** | | | | | **GPU** | |
| --- | --- | --- | --- | --- | --- | --- | --- | --- |
|  |  | **scTACL** | **SAVER** | **scImpute** | **MAGIC** | **scVGAE** | **scTACL** | **scVGAE** |
| frac0.1 | 7163 | 13320 | 3161 | 5914 | 1371 | 12719 | 14142 | 19480 |
| frac0.2 | 14327 | 15637 | 5629 | 21288 | 1352 | 20816 | 19302 | 38792 |
| frac0.3 | 21491 | 18714 | 8442 | 50923 | 1352 | 29463 | 26004 | 58929 |
| frac0.4 | 28655 | 22611 | 11022 | 89218 | 1356 | 23653 | 35848 | 70679 |
| frac0.5 | 38519 | 27269 | 13909 | 135451 | 1352 | - | 48498 | - |
| frac0.6 | 42982 | 32722 | 16369 | 184011 | 1348 | - | 55958 | - |
| frac0.7 | 50146 | 38955 | 19299 | 272918 | 1346 | - | 71794 | - |

Note: scVGAE could not be completed for subsampling fractions ≥ 0.5 due to excessive memory consumption exceeding the available server resources; therefore, no results are reported for these settings.

Table S9. Running Time under Different Fractions on Large-Scale Multi-Batch Datasets (S)

| **Batch** | **Number of Cells** | **scTACL** | **SAVER** | **scImpute** | **MAGIC** | **scVGAE** |
| --- | --- | --- | --- | --- | --- | --- |
| frac0.1 | 7163 | 31 | 13206 | 2880 | 39 | 158 |
| frac0.2 | 14327 | 78 | 26291 | 12226 | 27 | 402 |
| frac0.3 | 21491 | 136 | 39615 | 19124 | 53 | 875 |
| frac0.4 | 28655 | 230 | 56206 | 37126 | 42 | 1006 |
| frac0.5 | 38519 | 345 | 74375 | 69210 | 61 | - |
| frac0.6 | 42982 | 463 | 94974 | 105209 | 80 | - |
| frac0.7 | 50146 | 605 | 125332 | 167109 | 122 | - |

**Additional file 9: Spatial–Expression Relationship based on the Raw Count Matrix of the Liver Cancer Spatial transcriptomics dataset**

**
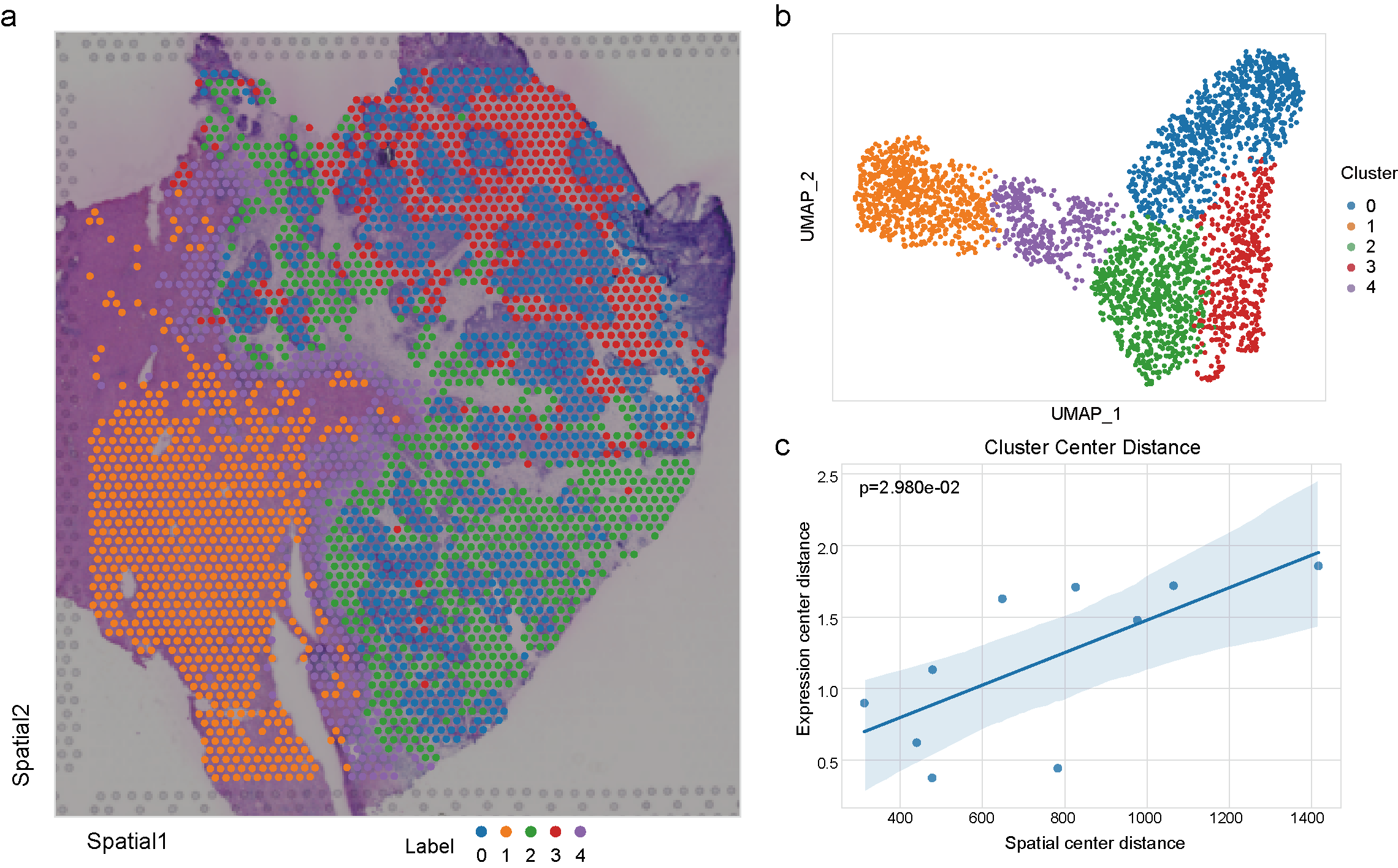
**

Figure S6. Spatial–expression relationship based on the raw count matrix of the liver cancer spatial transcriptomics dataset. (a) Spatial clustering results derived from the raw count matrix. (b) Uniform Manifold Approximation and Projection (UMAP) visualization of the data, with points colored by cluster assignment. (c) Correlation between spatial distance and expression distance across clusters.

**Additional file 10: Robustness and efficiency analysis of scTACL**

Table S10. Results of the Ablation Experiments

| **Dataset** | **NMI** | | | | | **ARI** | | | | | |  |
| --- | --- | --- | --- | --- | --- | --- | --- | --- | --- | --- | --- | --- |
|  | **scTACL** | **Without_CL** | **Without_ZINB** | **Without_CL&ZINB** | **Without_TD** | | **scTACL** | **Without_CL** | **Without_ZINB** | **Without_CL&ZINB** | **Without_TD** | |
| Adam | 0.8116 | 0.8239 | 0.8174 | 0.6832 | 0.8153 | | 0.7233 | 0.735 | 0.7295 | 0.4493 | 0.7307 | |
| Bach | 0.8099 | 0.8124 | 0.8119 | 0.7146 | 0.8092 | | 0.8016 | 0.8041 | 0.8036 | 0.5670 | 0.8009 | |
| Klein | 0.8599 | 0.8565 | 0.8522 | 0.8578 | 0.8568 | | 0.8469 | 0.8457 | 0.8437 | 0.8450 | 0.8462 | |
| Muraro | 0.8658 | 0.8628 | 0.8710 | 0.8085 | 0.8625 | | 0.9008 | 0.8967 | 0.9062 | 0.7106 | 0.8964 | |
| Plasschaert | 0.8107 | 0.7145 | 0.7143 | 0.6727 | 0.6730 | | 0.8035 | 0.5833 | 0.5803 | 0.5115 | 0.5170 | |
| Tosches_turtle | 0.7745 | 0.7737 | 0.7686 | 0.7716 | 0.7053 | | 0.5615 | 0.5631 | 0.5590 | 0.5611 | 0.5436 | |
| Young | 0.785 | 0.7623 | 0.7842 | 0.7123 | 0.6424 | | 0.6736 | 0.6627 | 0.6714 | 0.5772 | 0.4593 | |
| Pollen | 0.9268 | 0.9208 | 0.9280 | 0.9280 | 0.9057 | | 0.9133 | 0.8503 | 0.8838 | 0.8838 | 0.8920 | |
| Quake_10x_Bladder | 0.9453 | 0.8148 | 0.8146 | 0.5963 | 0.8127 | | 0.9737 | 0.7593 | 0.7588 | 0.4858 | 0.7584 | |
| Quake_10x_Limb_Muscle | 0.9906 | 0.9901 | 0.9893 | 0.7124 | 0.9908 | | 0.9953 | 0.9952 | 0.9946 | 0.5003 | 0.9959 | |
| Quake_10x_Spleen | 0.7978 | 0.7928 | 0.8043 | 0.5037 | 0.7914 | | 0.8203 | 0.8088 | 0.8349 | 0.3195 | 0.8027 | |
| Quake_Smart-seq2_Heart | 0.9425 | 0.8234 | 0.8166 | 0.8223 | 0.8194 | | 0.9692 | 0.6783 | 0.6727 | 0.6766 | 0.6771 | |
| Quake_Smart-seq2_Trachea | 0.7014 | 0.7035 | 0.6132 | 0.6993 | 0.7192 | | 0.5836 | 0.5705 | 0.5093 | 0.5747 | 0.5510 | |
| Quake_Smart-seq2_Diaphragm | 0.9705 | 0.9724 | 0.9687 | 0.8793 | 0.9622 | | 0.9868 | 0.9854 | 0.9845 | 0.9015 | 0.9754 | |
| Quake_Smart-seq2_Limb_Muscle | 0.9559 | 0.8198 | 0.8286 | 0.6892 | 0.8249 | | 0.9734 | 0.6574 | 0.6654 | 0.5234 | 0.6605 | |
| Quake_Smart-seq2_Lung | 0.8418 | 0.7437 | 0.7186 | 0.7193 | 0.7156 | | 0.8378 | 0.4994 | 0.4628 | 0.4665 | 0.4541 | |
| Romanov | 0.6781 | 0.6500 | 0.6265 | 0.5825 | 0.5825 | | 0.7063 | 0.6010 | 0.5970 | 0.5063 | 0.5072 | |
| Mean | 0.8511 | 0.8140 | 0.8075 | 0.7266 | 0.7935 | | 0.8277 | 0.7351 | 0.7328 | 0.5918 | 0.7099 | |
| Median | 0.8418 | 0.8148 | 0.8146 | 0.7124 | 0.8127 | | 0.8378 | 0.7350 | 0.7295 | 0.5611 | 0.7307 | |

Note: Without_CL indicates the ablation of the contrastive learning component; Without_ZINB indicates the removal of the ZINB-based reconstruction loss; Without_CL & ZINB indicates simultaneous removal of both components; Without_TD indicates the removal of the topological decoder.

**
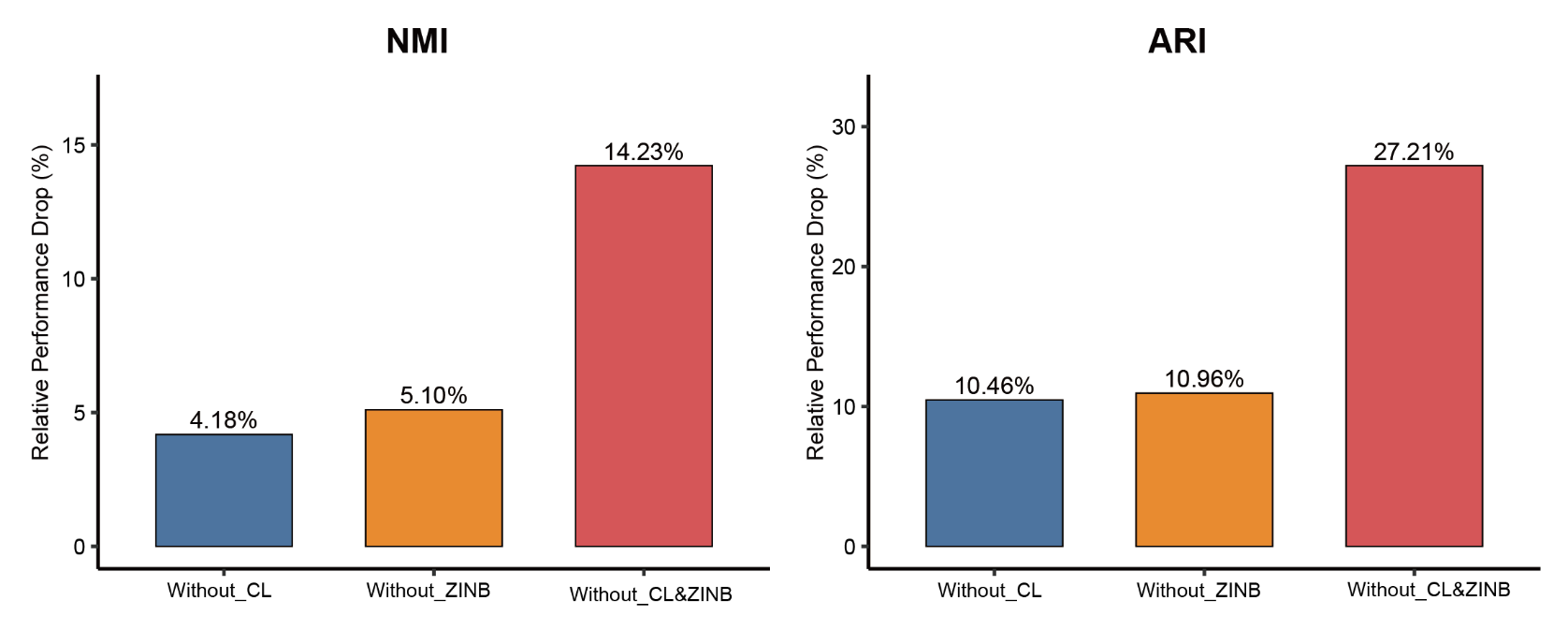
**

Figure S7. Ablation analysis of scTACL components on clustering performance

Table S11. Results of Experiments with Different Numbers of Highly Variable Genes

| **Dataset** | **NMI** | | | | **ARI** | | | |
| --- | --- | --- | --- | --- | --- | --- | --- | --- |
|  | **2000** | **3000** | **4000** | **5000** | **2000** | **3000** | **4000** | **5000** |
| Adam | 0.8151 | 0.8202 | 0.8417 | 0.8035 | 0.7328 | 0.7301 | 0.8191 | 0.7164 |
| Bach | 0.8027 | 0.8144 | 0.8089 | 0.8098 | 0.7976 | 0.8076 | 0.8021 | 0.8018 |
| Klein | 0.8588 | 0.8622 | 0.8555 | 0.863 | 0.8479 | 0.8484 | 0.8438 | 0.8499 |
| Muraro | 0.8668 | 0.8929 | 0.8791 | 0.8261 | 0.9031 | 0.9314 | 0.923 | 0.7168 |
| Plasschaert | 0.8085 | 0.8107 | 0.8019 | 0.796 | 0.8 | 0.8035 | 0.7814 | 0.7633 |
| Tosches turtle | 0.7688 | 0.7751 | 0.7534 | 0.7334 | 0.5594 | 0.5836 | 0.5245 | 0.4522 |
| Young | 0.7355 | 0.7899 | 0.7538 | 0.7615 | 0.6002 | 0.6767 | 0.636 | 0.6616 |
| Pollen | 0.9232 | 0.9298 | 0.9174 | 0.9121 | 0.9107 | 0.9133 | 0.9028 | 0.8995 |
| Quake 10x Bladder | 0.9423 | 0.9453 | 0.9442 | 0.9442 | 0.9721 | 0.9737 | 0.973 | 0.973 |
| Quake 10x Limb Muscle | 0.972 | 0.9919 | 0.9688 | 0.9852 | 0.9857 | 0.996 | 0.983 | 0.9932 |
| Quake 10x Spleen | 0.8413 | 0.8016 | 0.7817 | 0.7906 | 0.8978 | 0.8277 | 0.7895 | 0.8119 |
| Quake Smart-seq2 Heart | 0.9436 | 0.9494 | 0.9458 | 0.9398 | 0.9705 | 0.9736 | 0.9722 | 0.9691 |
| Quake Smart-seq2 Trachea | 0.6533 | 0.7041 | 0.5699 | 0.608 | 0.5481 | 0.5891 | 0.4987 | 0.516 |
| Quake Smart-seq2 Diaphragm | 0.9687 | 0.971 | 0.9551 | 0.9543 | 0.9845 | 0.9854 | 0.9784 | 0.9731 |
| Quake Smart-seq2 Limb Muscle | 0.9508 | 0.9593 | 0.9629 | 0.9532 | 0.9662 | 0.9738 | 0.9771 | 0.9708 |
| Quake Smart-seq2 Lung | 0.8329 | 0.8821 | 0.8039 | 0.8213 | 0.8212 | 0.8655 | 0.7381 | 0.7634 |
| Romanov | 0.675 | 0.6804 | 0.6741 | 0.6185 | 0.7039 | 0.7121 | 0.6967 | 0.6117 |
| Mean | 0.8447 | 0.8577 | 0.8364 | 0.8306 | 0.8236 | 0.8348 | 0.8141 | 0.7908 |
| Median | 0.8413 | 0.8622 | 0.8417 | 0.8213 | 0.8479 | 0.8484 | 0.8191 | 0.8018 |

Table S12. Results of Experiments with Different Numbers of Neighbor Nodes

| **Dataset** | **NMI** | | | | **ARI** | | | |
| --- | --- | --- | --- | --- | --- | --- | --- | --- |
|  | **3** | **4** | **5** | **6** | **3** | **4** | **5** | **6** |
| Adam | 0.7985 | 0.7994 | 0.8202 | 0.8026 | 0.7188 | 0.7183 | 0.7301 | 0.7224 |
| Bach | 0.8126 | 0.8115 | 0.8144 | 0.8121 | 0.8049 | 0.8027 | 0.8076 | 0.8042 |
| Klein | 0.8515 | 0.8557 | 0.8622 | 0.8524 | 0.8407 | 0.8447 | 0.8484 | 0.8406 |
| Muraro | 0.8241 | 0.8261 | 0.8929 | 0.8250 | 0.7004 | 0.7062 | 0.9314 | 0.7051 |
| Plasschaert | 0.8747 | 0.8067 | 0.8107 | 0.8078 | 0.9346 | 0.7983 | 0.8035 | 0.7976 |
| Tosches_turtle | 0.7761 | 0.7803 | 0.7751 | 0.7836 | 0.5700 | 0.5729 | 0.5836 | 0.5756 |
| Young | 0.7606 | 0.7463 | 0.7899 | 0.7497 | 0.6621 | 0.6367 | 0.6767 | 0.6385 |
| Pollen | 0.9179 | 0.9282 | 0.9298 | 0.9100 | 0.8998 | 0.9076 | 0.9133 | 0.8948 |
| Quake_10x_Bladder | 0.9794 | 0.9473 | 0.9453 | 0.9473 | 0.9921 | 0.9746 | 0.9737 | 0.9746 |
| Quake_10x_Limb_Muscle | 0.9866 | 0.9899 | 0.9919 | 0.9899 | 0.9937 | 0.9954 | 0.9960 | 0.9954 |
| Quake_10x_Spleen | 0.8393 | 0.8394 | 0.8016 | 0.8379 | 0.9007 | 0.9004 | 0.8277 | 0.8998 |
| Quake_Smart-seq2_Heart | 0.9430 | 0.9405 | 0.9494 | 0.9450 | 0.9697 | 0.9680 | 0.9736 | 0.9715 |
| Quake_Smart-seq2_Trachea | 0.6651 | 0.6705 | 0.7041 | 0.6655 | 0.5321 | 0.5371 | 0.5891 | 0.5378 |
| Quake_Smart-seq2_Diaphragm | 0.9729 | 0.9635 | 0.9710 | 0.9677 | 0.9863 | 0.9817 | 0.9854 | 0.9836 |
| Quake_Smart-seq2_Limb_Muscle | 0.9466 | 0.9541 | 0.9593 | 0.9541 | 0.9647 | 0.9666 | 0.9738 | 0.9666 |
| Quake_Smart-seq2_Lung | 0.8311 | 0.8601 | 0.8821 | 0.8427 | 0.8214 | 0.8475 | 0.8655 | 0.8350 |
| Romanov | 0.6732 | 0.6687 | 0.6804 | 0.6757 | 0.7043 | 0.6934 | 0.7121 | 0.6981 |
| Mean | 0.8502 | 0.8464 | 0.8577 | 0.8452 | 0.8233 | 0.8148 | 0.8348 | 0.8142 |
| Median | 0.8393 | 0.8394 | 0.8622 | 0.8379 | 0.8407 | 0.8447 | 0.8484 | 0.8350 |
